# Which are the most valued HIV pre-exposure prophylaxis attributes? A discrete choice experiment among sexual and gender minorities in Peru

**DOI:** 10.1371/journal.pone.0346154

**Published:** 2026-04-24

**Authors:** Oliver A. Elorreaga, Jazmin Qquellon, Kelika A. Konda, Karen E. Campos, Gino M. Calvo, Thiago S. Torres, Carlos F. Caceres, for the ImPrEP Study Group

**Affiliations:** 1 Universidad Peruana Cayetano Heredia, Centro de Investigación Interdisciplinaria en Sexualidad, SIDA y Sociedad, Lima, Peru; 2 Department of Population and Public Health Sciences, Keck School of Medicine, University of Southern California, Los Angeles, California, United States of America; 3 Instituto Nacional de Infectologia Evandro Chagas, Fundação Oswaldo Cruz (INI-Fiocruz), Rio de Janeiro, Brazil; University of Zimbabwe Faculty of Medicine: University of Zimbabwe College of Health Sciences, ZIMBABWE

## Abstract

**Background:**

There is potential demand for pre-exposure prophylaxis (PrEP) in Peru; however, little is known about preferences towards PrEP modalities or the importance of PrEP attributes. Our research focused on which attributes most influence PrEP modalities choice among sexual and gender minorities (SGM). We also assessed how participants’ preferences vary by recruitment strategy and population.

**Methods:**

We conducted a discrete choice experiment (DCE) in three mutually exclusive groups: (1) Implementation PrEP Project (ImPrEP) participants; (2) Sexually transmitted infections (STI) clinic attendees; and (3) Online respondents via social media outreach. PrEP attributes assessed were presentation, frequency of use, healthcare provider, HIV testing frequency, side effects, and efficacy. For the DCE, each participant was presented with 12 pairs of options with mixed attribute levels and had to choose one option per pair. The data were analyzed using choice-based conjoint analysis, employing Hierarchical Bayes estimation techniques to derive individual-level utility scores.

**Results:**

From June to October 2021, we recruited 2832 participants, with median age of 27 years (IQR = 22–33); 56.2% completed tertiary education; and 47.1% earned US$232 or less. In general, efficacy (27.5%, 95% CI 26.9–28.1), and frequency of use (21.3%, 95%CI 20.8–21.7) were the most important attributes, with the online respondents the highest importance on efficacy (31.9%, 95%CI 31.0–32.8). Concerned about potential side effects were more common among STI clinic attendees (17.5% [95%CI: 16.9–18.2]) and online respondents (18.8% [95%CI: 18.3–19.3]), while the ImPrEP participants placed the greatest importance on the frequency of use [26.7%, 95%CI 25.6–27.7].

**Conclusions:**

Overall, attributes such as efficacy and frequency of use were the most important PrEP attributes across the recruitment strategies; however, current ImPrEP participants placed more weight on frequency than the other groups. Our data may be helpful for future PrEP scale-up strategies, anticipating potential users concerns, and learning from current users.

## Introduction

Between 2010 and 2022, the incidence of HIV infection in Latin America has shown unsubstantial changes compared to other regions of the world. In 2022, 120,000 (95% CI: 94,000 - 130,000) people newly acquired HIV, and 2.2 million (95% CI: 2.0 - 2.5) were living with HIV in Latin America [1]. In addition, HIV in Latin America is concentrated among gay, bisexual and other men who have sex with men (MSM), with a median prevalence of 9.5% (IQR: 8.1% - 16.9%) and transgender women (TW) with a median prevalence of 15.5% (IQR: 8.7% - 30.8%) [1,2]. In Peru, a 31% increase in HIV incidence per 1000 uninfected population has been observed since 2010 [2]. In 2022, the estimated HIV prevalence for MSM and TW was 10% among MSM and 30.9% among TW [3]. The higher prevalence of HIV among key populations reveals the need to improve the public HIV prevention strategy. Pre-exposure prophylaxis (PrEP) is a highly effective prevention strategy that reduces the risk of HIV infection [4].

In 2010, the Preexposure Prophylaxis Initiative (iPrEx) study was the first to demonstrate the efficacy of oral PrEP with emtricitabine and tenofovir disoproxil fumarate (FTC/TDF) [5]. iPrEx was conducted mainly in South American countries (Brazil, Ecuador, and Peru) and showed that oral PrEP reduced HIV incidence by 44%; however, there was poor adherence to the daily pill regimen. In addition, statistical models in iPrEx of the relationship between drug adherence levels and TDF efficacy have reported that an adherence of 7 doses per week corresponded to 99% efficacy, 4 doses per week to 96%, and 2 doses per week to 76% [6]. More recently, the largest multicenter Implementation PrEP Project (ImPrEP) in Latin America, reported the feasibility of same-day oral PrEP delivery within the health systems of Brazil, Mexico, and Peru, with a low proportion of early loss to follow-up and higher adherence in Brazil and Mexico, but a higher proportion of loss and lower adherence in Peru [7]. The impact of PrEP use among MSM has also been studied in Latin America. A modeling study in Brazil showed that a reduction in HIV incidence of approximately 30% could be achieved in 5 years if 60% of MSM were to take PrEP within 24 months [8]. Based on the evidence of the effectiveness of PrEP, the World Health Organization (WHO) recommends that MSM and TW engaging in HIV high-risk behavior should be reached by PrEP services [9]. In 2022, PrEP was available as public policy in 10 Latin American countries [10], with Brazil being the first country in the region to provide PrEP free of charge through its public health system for populations at substantial risk for HIV infection [11]. In 2023, Peru has begun distributing PrEP within its public health system to key populations [12].

The most frequently used PrEP modality is daily oral PrEP, taking one oral pill of FTC/TDF or tenofovir alafenamide fumarate (TAF)/FTC daily. Event driven PrEP (ED-PrEP also known as PrEP 2-1-1), is another regimen or oral PrEP and consists of taking pills before and after sexual contact (two pills of FTC/TDF between 2 and 24 hours before sexual intercourse, then one pill 24 hours after the initial pill, and finally another pill 24 hours after the third pill) [13]. The use of both oral PrEP regimens remains a challenge due to adherence. The most significant barriers are remembering to take the pill consistently, non-routinization, and the risk of unplanned sex [14]. For this reason, PrEP has been developed in alternative presentations, such as long-acting injectables based on nanoformulations of cabotegravir or rilpivirine, which can be administered every 1, 2, or 3 months [14,15]. Particularly, long-acting injectable cabotegravir has been shown to be superior in preventing HIV infection compared to daily oral PrEP among MSM and TW [16]. The WHO currently recommends this modality of PrEP as an alternative to pills [17]. In addition, the PURPOSE 1 clinical trial demonstrated the efficacy of injectable lenacapavir, administered every six months, as PrEP among adolescents and young women, showing a 100% reduction in HIV incidence compared to daily oral FTC/TDF PrEP [18]. Meanwhile, the PURPOSE 2 study, which is evaluating the efficacy of lenacapavir among MSM, TW, and non-binary individuals, has reported interim results indicating a 96% reduction in HIV incidence [19]. Furthermore, subcutaneous implants with TDF derivatives are a potential new technology that is being evaluated for yearly administration. Promising advantages of subcutaneous implants include more consistent drug release and easy removal in case of toxicity [20].

Because of the variety in PrEP regimens, studies around the world have continued to assess the preferences of participants in different modes of taking PrEP [21–25]. Pereira et al. introduced the assessment of PrEP preferences in Brazil using a discrete choice experiment (DCE), which helped identify key PrEP attributes and their levels to enhance future PrEP uptake and adherence [26]. The goal is to optimize PrEP for users and improve the design of health policies for HIV prevention. However, in Latin America, there are very few studies that have explored the assessment that potential and/or actual users have of the different PrEP modalities. Therefore, the present project aimed to assess the preferences for potential PrEP delivery programs in a Peruvian setting among sexual and gender minorities (SGM) using a DCE. Additionally, we sought to understand how preferences for attributes and levels of PrEP varied by recruitment strategies.

## Materials and methods

### Study population and procedures

We conducted a cross-sectional survey with a DCE between June 1 and October 31, 2021, among SGM such as MSM, TW, non-binary, and others. Eligible participants were aged at least 18 years, self-reported a non-HIV positive status, and could provide informed consent in electronic form. Participants were recruited through different invitation channels and formed three exclusive groups: 1. ImPrEP participants; 2. Sexually transmitted infections (STI) clinic users; and 3. Online respondents.

The first group included new, ongoing and former participants of the ImPrEP project, a multicenter project for the implementation of PrEP in Brazil, Mexico, and Peru [7,27]. These participants were recruited at nine ImPrEP-affiliated sites in Peru, including five in Lima and Callao and four in other regions (Pucallpa, Ica, Chimbote, and Trujillo). The second group comprised individuals who attended the same STI clinics for reasons unrelated to the ImPrEP project, such as counseling, HIV or other STI testing, receiving treatment, and others. These clinics were public health facilities and community-based organizations that provide STI/HIV services in coordination with the Peruvian Ministry of Health and were selected based on their role as ImPrEP sites and their accessibility to key populations.

The third group was recruited online, through social media and peer networks, using dating apps (Grinder and Hornet), social media platforms (Facebook, WhatsApp, and Instagram) and other channels (local networks, word of mouth). The selection of participants in the three groups was non-probabilistic by convenience, which may limit generalizability but allowed access to diverse and hard-to-reach subpopulations.

Participants in the first and second groups were invited while in the waiting room at STI clinics. They were asked to complete the computer-based survey with the assistance of a trained staff, as needed. Participants were classified based on the reason for attending the clinic: individuals enrolled in the ImPrEP project were classified as ImPrEP participants, while those attending for other services were classified as STI clinic users. The third group was redirected to the online survey when they clicked on a project advertisement or invitation. Participants were classified based on their recruitment source, regardless of their PrEP use or recent clinic visits. Because STI clinics and ImPrEP participants were recruited exclusively in person, no overlap between them and the online group was possible. To maintain mutual exclusivity among groups, we excluded online respondents who reported currently using PrEP through the ImPrEP project, as well as individuals who indicated that they previously completed the survey. The aim of having three groups was to compare the DCE among experienced individuals (ImPrEP participants), those seeking health services (STI clinic users), and those not necessarily seeking HIV prevention services (online respondents).

### Discrete choice modeling

The design of the DCE was adapted from the study performed by Pereira et al., who identified and validated the most important attributes of PrEP adherence through a literature review and in-depth interviews with HIV healthcare professionals, MSM and TW users, and non-users of PrEP [26,28]. We considered the following six PrEP attributes: (1) presentation, (2) frequency of use, (3) healthcare provider, (4) HIV testing frequency, (5) side effects, and (6) efficacy. Details of the attributes and their levels are described in S1 Table. Additionally, specific rules were implemented to prevent the emergence of irrelevant or unrealistic combinations of attributes. For example:

- Oral PrEP (pills) was only compatible with usage frequencies of “once a day” and “before sexual intercourse.” Conversely, Injectable PrEP was only compatible with “once a month” and “once every 3 months.” Implant PrEP was only combined with “once every 3 months” and “once every six months.” These restrictions avoided unrealistic scenarios such as “oral PrEP” with use “once every 6 months.”
- Regarding health service visit frequencies, Oral PrEP (pills) could only be combined with “visits once every 3 months,” while a “once every 6 months” visit frequency was not permitted. Inversely, implant PrEP was linked to “visits every 6 months for check-ups,” and “visits every 3 months” were not feasible. Injectable PrEP allowed flexibility with both types of health center visit frequencies (every 3 or 6 months).
- For the attributes “PrEP usage frequency” and “health service visit frequency,” high-frequency PrEP usage (such as daily usage, usage every time before sexual intercourse (ED PrEP), once a month, and once every 2 or 3 months) could not be paired with infrequent health service visits (specifically “visits once every 6 months”). Conversely, low-frequency PrEP usage (specifically “once every 6 months”), which involves less frequent dosing and potentially longer-acting formulations, was only viable with health service visits scheduled every 6 months.
- Concerning Side effects, the attribute level indicating “mild side effects that last for weeks” could not be mixed with the “use before at-risk sexual intercourse” (ED PrEP) frequency. However, enduring side effects were combinable with all other levels of usage frequency: “daily PrEP,” “once a month,” “once every 2/3 months,” and “once every 6 months,” which was more consistent.
- Finally, the Cost of PrEP at the level “Free but with longer waiting time” was only combinable with “Public sector provider,” while “Higher cost and shorter waiting time” could only be combined with “Provided by the private sector (clinics)” and “Provided by an NGO.”

Based on these rules, we included 12 pairs of hypothetical scenarios of PrEP presentations with different combinations of their attribute levels, which were randomly assigned to participants. Each participant completed a single block of 12 choice tasks, selecting only one option per pair (Option A or Option B). To enhance design variability, the Sawtooth software automatically generated unique combinations of tasks for each participant, resulting in several hundred distinct versions of the choice-based conjoint (CBC) questionnaire. Blocking, randomization and generation of efficient attribute-level combination were implemented automatically by the software, and no additional design restrictions were applied.

We also collected information about sociodemographic characteristics, such as age (categorized as 18-24 and ≥ 25 years old); gender identity (cisgender men and transgender/non-binary/other); level of education (completed secondary or less, and higher education); personal income per month (minimum wage [US $232] or less, and more than the minimum wage); district of residence; and race (white, mixed-race, and Asian/black/indigenous/other).

In addition, we asked about sexual behavior variables, such as condomless anal intercourse (CAI), number of sexual partners, chemsex, and engagement in sexual work, referring to experiences within the last six months. We also assessed HIV risk perception based on participants’ self-assessment (dichotomized as low/moderate [none, low, and some risk] and high [high risk/certainty of infection]), and calculated the HIV Incidence Risk Index for SGM (HIRI-SGM), which considers scores of 10 or higher as “high risk”. The HIRI-SGM index has been previously used in Latin America [29]. We further asked about prior experience with PrEP, defined as any current or past use, and not restricted to a specific timeframe. The completed survey is available in Supporting Information S2 File.

### Statistical analysis

We described frequencies and percentages of sociodemographic, sexual behavior, and variables related to PrEP for each recruitment group. Differences across recruitment groups were assessed using chi-square tests for categorical variables and Kruskal-Wallis tests for numerical variables. Differences in PrEP attributes were also described according to sociodemographic and behavioral characteristics. All the analyses were performed using STATA v.17.

Discrete choice data were analyzed using Sawtooth Software’s Lighthouse Studio 9.11.0, employing the CBC analysis module. This approach simulates decision-making by presenting respondents with sets of alternative PrEP modalities and recording their choices. The CBC method naturally accounts for the Independence of Irrelevant Alternatives (IIA) through its design and allows for a detailed assessment of trade-offs that participants are willing to make between different attributes of PrEP modalities.

Hierarchical Bayes (HB) estimation techniques were used to derive individual-level utility scores for each attribute level associated with PrEP modalities. The HB estimation used 20,000 iterations with a burn-in period of 10,000 iterations. Convergence was assessed using the Root Mean Square (RMS) standard error of the mean, with convergence considered achieved when RMS values stabilized below 0.05. HB estimation stabilizes individual-level estimates by pooling information across respondents, enhancing the accuracy and reliability of the utility estimates across a diverse study population.

The analysis focused on estimating the relative importance of each PrEP attribute and exploring how these preferences varied across different demographic segments within the study population. Part-worth utilities represent the relative value that respondents assign to each attribute level, measured on an interval scale where higher values indicate greater preference. Utility scores were zero-centered within each attribute such that positive values indicate above-average preference and negative values indicate below-average preference relative to the other levels of the same attribute. The magnitude of utility differences between attribute levels quantifies the strength of preference for one over another.

### Ethics

This study was approved by the Ethical Review Committee at Universidad Peruana Cayetano Heredia, with study number SIDISI 204463.

## Results

### Study participants

**Fig 1:**
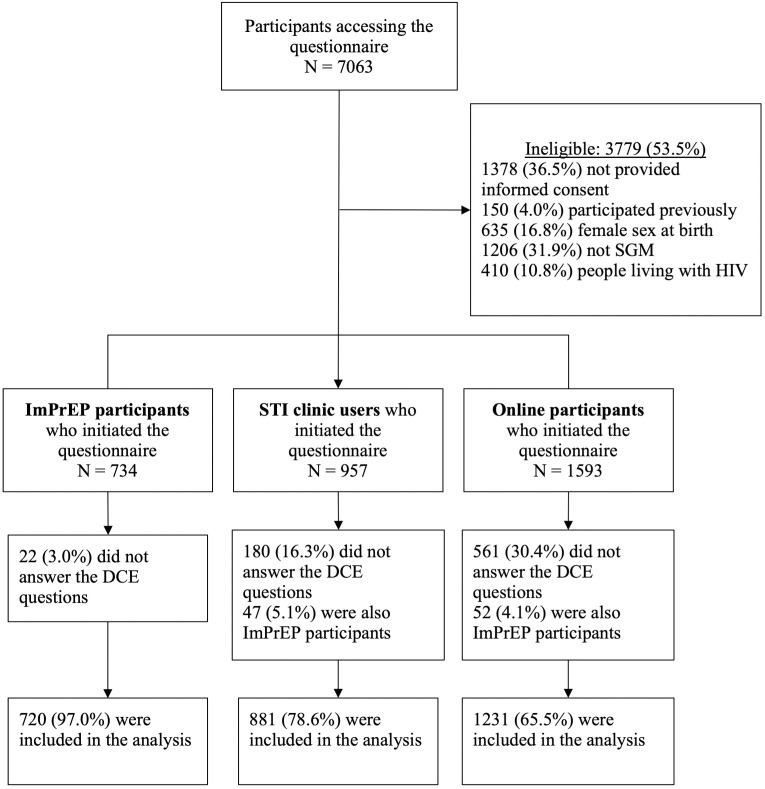
Study flowchart of participant recruitment and selection. The flowchart shows the progression from initial questionnaire access (N = 7063) through eligibility screening to the final analytical sample (N = 2832) distributed across three recruitment groups: ImPrEP participants (n = 720), STI clinic users (n = 881), and online participants (n = 1231). Reasons for ineligibility and non-completion of the Discrete Choice Experiment (DCE) questions are detailed for each group.

Among the 7063 individuals who accessed the questionnaire, 3779 (53.5%) were ineligible because 36.5% did not provide informed consent, 4.0% had participated previously, 16.8% were female sex at birth, 31.9% did not self-identify as SGM and 10.8% were people living with HIV. Of the 3284 (46.5%) eligible participants, 99 (3.0%) individuals were excluded because they belonged to two recruitment groups. Finally, 2832 (86.2%) answered the DCE questions and were included in the analysis (see Fig 1). We enrolled 720 (24.5%) ImPrEP participants, 881 (31.1%) users of STI clinics, and 1231 (43.5%) online respondents. Facebook (60.7%) was the main source of online recruitment, followed by Grindr (15.3%), WhatsApp (11.4%), Instagram (7.9%), Hornet (0.2%), and others (4.5%).

### Sociodemographic and behavioral characteristics

Table 1 showed sociodemographic and behavioral characteristics. The median age was 27 (Interquartile range [IQR]: 23–33) years, most of the participants self-identified as MSM (85.6%) and resided in the capital of the department (86.6%), 56.2% had more than secondary education level and 47.1% receiving one minimum wage or less per month. In the last 6 months, 74.5% reported having had CAI, 37.0% had had more than five sexual partners, and 11.6% had chemsex. Being at high risk for HIV acquisition was perceived by 23.6% of participants, while 66.1% were at high risk according to the HIRI-SGM index. One-third of individuals had previous experience using PrEP and 69.8% were aware of it; however, only 25.8% were aware of ED-PrEP.

**Table 1:** Socio-demographic and behavioral characteristics among SGM by recruitment strategy groups.

Characteristics	Total N = 2832, (%)	ImPrEP participants N = 720, (%)	STI clinic users N = 881, (%)	Online respondents N = 1231, (%)	*p* value
**Gender Identity**					<0.001
Cisgender men	2424 (85.6)	622 (86.4)	672 (76.3)	1130 (91.8)	
Trans/non-binary/other	408 (14.4)	98 (13.6)	209 (23.7)	101 (8.2)	
**Age in years (Median [p25-p75])**	27 (23-33)	29 (25-35)	27 (23-33)	25 (21-31)	<0.001
**Age in years**					<0.001
18 - 24	1031 (36.4)	163 (22.6)	297 (33.7)	571 (46.4)	
≥ 25	1801 (63.6)	557 (77.4)	584 (66.3)	660 (53.6)	
**Race**					0.049
White	332 (11.7)	76 (10.5)	97 (11.0)	159 (12.9)	
Mixed-race	2110 (74.5)	555 (77.1)	673 (76.4)	882 (71.7)	
Black/Asian/Indigenous/Other	390 (13.8)	89 (12.4)	111 (12.6)	190 (15.4)	
**Residence in the capital**					0.020
No	381 (13.4)	81 (11.3)	110 (12.5)	190 (15.4)	
Yes	2451 (86.6)	639 (88.7)	771 (87.5)	1041 (84.6)	
**Education level**					<0.001
≤ secondary education	1241 (43.8)	246 (34.2)	544 (61.7)	451 (36.6)	
> secondary education	1591 (56.2)	474 (65.8)	337 (38.3)	780 (63.4)	
**Monthly income**					<0.001
≤ minimum wage	1221 (47.1)	261 (39.3)	430 (51.7)	530 (48.3)	
> minimum wage	1372 (52.9)	404 (60.7)	401 (48.3)	567 (51.7)	
**Prior experience with PrEP**					<0.001
No	2029 (71.6)	12 (1.7)	841 (95.5)	1176 (95.5)	
Yes (currently or previously)	803 (28.4)	708 (98.3)	40 (4.5)	55 (4.5)	
**PrEP awareness**					<0.001
No	855 (30.2)	24 (3.3)	495 (56.2)	336 (27.3)	
Yes	1977 (69.8)	696 (96.7)	386 (43.8)	895 (72.7)	
**CAI** ^**a**^					<0.001
No	640 (23.4)	121 (16.9)	162 (18.6)	357 (30.9)	
Yes	2040 (74.5)	584 (81.4)	678 (78.0)	778 (67.5)	
Didn't answer	59 (2.1)	12 (1.7)	29 (3.3)	18 (1.6)	
**Number of sexual partners** ^a^					<0.001
0 - 1	616 (22.7)	126 (17.7)	146 (17.1)	344 (30.1)	
2 - 5	1091 (40.2)	258 (36.2)	389 (45.5)	444 (38.8)	
> 5	1004 (37.0)	329 (46.1)	320 (37.4)	355 (31.1)	
**Chemsex** ^**a**^					0.228
No	2387 (88.4)	639 (89.9)	735 (87.1)	1013 (88.5)	
Yes	312 (11.6)	72 (10.1)	109 (12.9)	131 (11.5)	
**Sex worker** ^**a**^ **(n = 2669)**					<0.001
No	2102 (78.8)	559 (79.2)	564 (67.3)	979 (87.0)	
Yes	567 (21.2)	147 (20.8)	274 (32.7)	146 (13.0)	
**HIV risk perception**					<0.001
Low/moderate	2051 (76.4)	456 (64.7)	634 (74.5)	961 (85.1)	
High	634 (23.6)	249 (35.3)	217 (25.5)	168 (14.9)	
**HIRI-SGM** ^a^					<0.001
Low risk	959 (33.9)	170 (23.6)	263 (29.8)	526 (42.7)	
High risk	1873 (66.1)	550 (76.4)	618 (70.2)	705 (57.3)	

PrEP: pre-exposure prophylaxis.

CAI: Condomless anal intercourse.

HIRI-SGM: HIV Incidence Risk Index for sexual and gender minorities.

^a^ in the last 6 months.

*p* values represent statistical comparison across the three recruitment groups using chi-square tests for categorical variables and Kruskal-Wallis tests for continuous variables.

### Relative Importance of PrEP Attributes

Table 2 showed the relative importance of PrEP attributes in the total sample and by recruitment group. Overall, the most important attributes were efficacy (27.5% [95% CI: 26.9% – 28.1%]) and frequency of use (21.3% [95% CI: 20.8% – 21.7%]). Among the ImPrEP participants, the greatest relative importance was frequency of use (26.7% [95% CI: 25.6% – 27.7%]), followed by efficacy (23.9% [95% CI: 22.7% – 25.1%]), and the presentation of PrEP (15.2% [95% CI: 14.6% – 15.8%]). For STI clinic attendees, the highest importance was placed on efficacy (24.4% [95% CI: 23.4% – 25.4%]), followed by frequency of use (21.2% [95% CI: 20.5% – 21.9%]), and side effects (17.5% [95% CI: 16.9% – 18.2%]. Among online respondents, efficacy had the highest relative importance (31.9% [95% CI: 31.0% – 32.8%]), followed by side effects (18.8% [95% CI: 18.3% – 19.3%]).

**Table 2:** Relative importance of attributes of PrEP for each group of participants.

Attribute	Total (%, 95 CI)	ImPrEP participants (%, 95 CI)	STI clinic users (%, 95 CI)	Online respondents (%, 95 CI)
Presentation	14.4 (14.1 - 14.7)	15.2 (14.6 - 15.8)	16.0 (15.4 - 16.6)	12.8 (12.3 - 13.2)
Frequency of use	21.3 (20.8 - 21.7)	26.7 (25.6 - 27.7)	21.2 (20.5 - 21.9)	18.2 (17.7 - 18.7)
Provider	8.9 (8.7 - 9.1)	9.0 (8.6 - 9.4)	9.0 (8.7 - 9.4)	8.7 (8.4 - 9.0)
Frequency of visits for HIV testing	10.7 (10.4 - 11.0)	11.2 (10.7 - 11.8)	11.8 (11.3 - 12.4)	9.6 (9.2 - 10.0)
Side effects	17.2 (16.8 - 17.5)	14.0 (13.3 - 14.6)	17.5 (16.9 - 18.2)	18.8 (18.3 - 19.3)
Efficacy	27.5 (26.9 - 28.1)	23.9 (22.7 - 25.1)	24.4 (23.4 - 25.4)	31.9 (31.0 - 32.8)

Note: The relative importance of attributes sums to 100%. These percentages are derived from the utility scores presented in Table 3, where larger utility ranges indicate stronger preferences between attribute levels.

### Preferences for PrEP Attribute Levels

Table 3 showed preferences for specific levels of attributes (utility). Overall, participants preferred 90% efficacy (utility: 76.7), and a daily frequency of use (utility: 25.4). Among ImPrEP participants, the most preferred level were daily use (utility: 60.1), 90% efficacy (utility: 65.1) and implant presentation (utility: 28.4). For STI clinic attendees, the most preferred levels were 90% efficacy (utility: 65.1), daily use (utility: 19.1), and no side effects (utility: 48.1). Among online respondents, the most preferred levels were 90% efficacy (utility: 91.9) and no side effects (utility: 54.5).

**Table 3:** Zero-centered part-worth utilities for PrEP attribute levels by recruited groups.

Attribute	Total (n = 2832)	ImPrEP participants (n = 720)	STI clinic users (n = 881)	Online respondents (n = 1231)
Presentation				
Oral pill	−21.05	−22.09	−22.55	−19.38
Injectable (intramuscular)	−1.46	−6.30	0.82	−0.25
Implant (subcutaneous)	22.51	28.39	21.73	19.63
Frequency of use				
Daily	25.38	60.07	19.12	9.57
Event-Driven	−11.58	−26.48	−7.53	−5.76
Monthly	7.42	6.91	6.42	8.42
Once per trimester	11.50	9.72	9.34	14.08
Once per semester	−32.72	−50.22	−27.34	−26.32
Provider				
Public healthcare centers	4.83	8.31	4.68	2.90
Private healthcare centers	−18.78	−21.18	−17.86	−18.04
Non-Governmental Organization	13.95	12.87	13.18	15.14
Frequency of visits for HIV testing				
Once per trimester	16.85	11.09	23.33	15.58
Once per semester	−16.85	−11.09	−23.33	−15.58
Side-effects				
None	47.98	36.76	48.09	54.47
Mild and disappear in the first weeks	−0.48	−1.04	−0.95	0.19
Mild and last for weeks	−47.50	−35.72	−47.14	−54.66
Efficacy				
90% (9 in 10 remain HIV negative)	76.73	65.09	65.06	91.89
80% (8 in 10 remain HIV negative)	0.44	−0.06	0.38	0.78
70% (7 in 10 remain HIV negative)	−77.17	−65.03	−65.44	−92.67

Interpretation: negative values imply negative preferences for the program attribute and the magnitude of the preference is associated with the strength.

### Subgroup analysis by sociodemographic characteristics

According to the segmentation group by sociodemographic characteristics (see Table 4), we found that the 18–24 years old group gave slightly more importance to efficacy (29.5% [95% CI: 28.5% – 30.5%]), and side effects (18.3% [95% CI: 17.7% – 18.9%]) than the older age group. Meanwhile, those aged > 24 were more focused on the frequency of PrEP use (22.4% [95% CI: 21.9% – 23.0%]), and presentation (14.9% [95% CI: 14.5% – 15.3%]) than younger participants. In addition, participants living in the capital city gave slightly less importance to the frequency of use (20.8% [95% CI: 20.4% – 21.3%]), and they were more concerned about potential side effects (17.7% [95% CI: 17.3% – 18.0%]) than participants from outside the capital.

**Table 4:** Relative importance of attributes of PrEP according to sociodemographic characteristics.

	Gender Identity		Age (years)		Residence in the capital	
	Cisgender men	Trans/non-binary/other	18 - 24	≥ 25	No	Yes
	(%, 95 CI)	(%, 95 CI)	(%, 95 CI)	(%, 95 CI)	(%, 95 CI)	(%, 95 CI)
Presentation	14.2 (13.9 - 14.6)	15.5 (14.7 - 16.3)	13.5 (13.0 - 14.0)	14.9 (14.5 - 15.3)	13.2 (12.4 - 14.0)	14.6 (14.3 - 14.9)
Frequency of use	21.1 (20.6 - 21.5)	22.4 (21.3 - 23.6)	19.2 (18.6 - 19.8)	22.4 (21.9 - 23.0)	24.0 (22.7 - 25.3)	20.9 (20.4 - 21.3)
Provider	8.8 (8.6 - 9.0)	9.4 (8.8 - 9.9)	9.0 (8.7 - 9.3)	8.8 (8.5 - 9.0)	9.0 (8.5 - 9.6)	8.8 (8.6 - 9.0)
Frequency of visits for HIV testing	10.7 (10.4 - 11.0)	11.1 (10.3 - 11.9)	10.4 (10.0 - 10.9)	10.9 (10.5 - 11.3)	11.2 (10.4 - 12.0)	10.6 (10.3 - 10.9)
Side effects	17.3 (16.9 - 17.7)	16.4 (15.5 - 17.4)	18.3 (17.7 - 18.9)	16.6 (16.1 - 17.0)	14.2 (13.3 - 15.1)	17.7 (17.3 - 18.0)
Efficacy	27.9 (27.3 - 28.6)	25.2 (23.7 - 26.8)	29.5 (28.5 - 30.5)	26.4 (25.6 - 27.2)	28.3 (26.6 - 29.9)	27.4 (26.8 - 28.1)

Note: The relative importance of attributes sums to 100%.

### Subgroup analysis by behavioral characteristics

According to the segmentation group by behavioral characteristics (see Table 5), we found that participants who reported chemsex were more concerned about side effects (18.3% [95% CI: 17.2% – 19.4%]) but less concerned about the frequency of use (19.5% [95% CI: 18.4% – 20.7%]) than those who do not reported chemsex. Sex workers showed less concern about the rate of efficacy (25.6% [95% CI: 24.3% – 27.0%]); meanwhile, they gave slightly more importance to the PrEP presentation (15.3% [95% CI: 14.6% – 16.0%]) than the non-sex worker group. Additionally, we found that the group at higher risk of acquiring HIV (according to HIRI-SGM index) gave greater importance to the frequency of PrEP use (22.1% [95% CI: 21.6% – 22.7%]) and lower importance to the side effects (16.5% [95% CI: 16.1% – 17.0%]) and efficacy rate (26.9% [95% CI: 26.2% – 27.7%] than the group with a lower risk of HIV infection.

**Table 5:** Relative importance of attributes of PrEP according to behavioral characteristics.

	HIRI-GSM		Chemsex		Sex worker	
	Low risk	High risk	No	Yes	No	Yes
	(%, 95 CI)	(%, 95 CI)	(%, 95 CI)	(%, 95 CI)	(%, 95 CI)	(%, 95 CI)
Presentation	13.9 (13.4 - 14.4)	14.7 (14.3 - 15.0)	14.4 (14.1 - 14.7)	14.8 (13.8 - 15.8)	14.2 (13.9 −14.6)	15.3 (14.6 - 16.0)
Frequency of use	19.6 (19.0 - 20.3)	22.1 (21.6 - 22.7)	21.6 (21.2 - 22.1)	19.5 (18.4 - 20.7)	21.3 (20.8 - 21.8)	21.9 (21.0 - 22.8)
Provider	8.6 (8.3 - 9.0)	9.0 (8.7 - 9.2)	8.9 (8.6 - 9.1)	9.1 (8.5 - 9.7)	8.8 (8.6 - 9.1)	9.0 (8.5 - 9.4)
Frequency of visits for HIV testing	10.6 (10.2 - 11.1)	10.8 (10.4 - 11.1)	10.8 (10.5 - 11.1)	10.5 (9.6 - 11.4)	10.6 (10.3 - 10.9)	11.4 (10.7 - 12.0)
Side effects	18.5 (17.9 - 19.1)	16.5 (16.1 - 17.0)	16.9 (16.6 - 17.3)	18.3 (17.2 - 19.4)	17.2 (16.8 - 17.6)	16.8 (16.0 - 17.6)
Efficacy	28.7 (27.7 - 29.8)	26.9 (26.2 - 27.7)	27.4 (26.7 - 28.1)	27.8 (25.9 - 29.6)	27.8 (27.1 - 28.5)	25.6 (24.3 - 27.0)

Note: The relative importance of attributes sums to 100%.

## Discussion

This study utilized a discrete choice experiment to elucidate preferences for PrEP attributes among SGM in Peru. We found a marked preference for high efficacy and regular frequency of use, particularly for 90% efficacy and daily PrEP. While these preferences were consistent across all groups, including ImPrEP participants, attendees at STI clinics, and individuals recruited online, there were notable differences in the relative importance placed on other attributes and by specific subgroups. These findings align with prior research, highlighting efficacy as a critical concern among sexual and gender minorities [22,26,30–32].

Our study identified that younger participants and cisgender MSM emphasized efficacy in their PrEP preferences, consistent with findings from global research [22,26,30,31]. However, unlike observations in studies such as Pereira et al. [26], our research revealed distinct preferences in PrEP attributes between online and on-site recruitment groups. A heightened concern for side effects was evident among younger individuals, particularly those residing in urban areas and engaging in chemsex. This discrepancy may be due to differences in baseline knowledge and experience with PrEP. In Brazil, Pereira et al., reported a rate of 55% of current or previous PrEP use in the on-site groups, while the online group reached a rate of 29% [26]. This indicates higher knowledge and less divergent experience with PrEP across groups compared to our Peruvian sample, where current or prior experience in the on-site groups was 93% (ImPrEP participants: 98.3%; STI clinic users: 4.5%), and only 4.5% among online respondents. These differences could suggest that awareness and experience with PrEP significantly impact attribute preferences, particularly regarding side effects.

This demographic’s specific concerns underscore the need for targeted communication strategies, utilizing key platforms such as social networks and dating apps, to effectively disseminate accurate information about PrEP. Such tailored strategies are crucial to improve PrEP acceptability and adherence [33–35]. Additionally, the pronounced importance given to PrEP presentation, especially among sex workers, highlights the necessity for more personalized PrEP delivery methods. These methods should be carefully designed to align with the diverse lifestyles and risk profiles of individuals within these key populations.

Despite Peru's policy of providing free PrEP access to key populations, our study, which collected data prior to the public access to PrEP in Peru, suggests potential gaps in policy implementation and real-world user preferences that could enhance the current implementation. While there was a strong preference for daily PrEP use potentially due to its current availability, the interest in less frequent use modalities, such as once per trimester dosing, may reflect underlying challenges in the healthcare system, including the burden of frequent healthcare visits and economic constraints in Peru. This is echoed in the findings of Chakrapani et al., where a preference for on-demand PrEP in India was linked to cost considerations [30], highlighting the impact of economic factors on PrEP preferences. These insights underscore the need for healthcare reforms in Peru that focus on patient convenience and flexibility, and for adaptable PrEP strategies that consider both economic affordability and healthcare accessibility in diverse settings.

Different recruitment groups in our study showed varying preferences for PrEP attributes. While STI clinic attendees and online respondents exhibited a heightened concern for side effects, ImPrEP participants prioritized the frequency of use. Additionally, online respondents prioritized the efficacy in their PrEP preferences compared to the other recruitment groups. This could reflect that people not linked to services have different knowledge and preferences, highlighting the need for targeted campaigns to increase awareness of PrEP. This diversity suggests that public health messaging and PrEP delivery strategies in Peru need to be tailored to address these specific concerns and preferences. Furthermore, promoting campaigns to increase PrEP awareness is essential.

The relative importance weights derived from our DCE provide actionable insights for PrEP implementation in Peru. Evidence from external validity studies indicates that preferences elicited through DCE can reliably predict health-related behavior and inform evidence-based resource allocation [36]. The high relative importance assigned to efficacy (27.5%) and frequency of use (21.3%) suggests that communication strategies should emphasize demonstrated effectiveness, while implementation planning should consider the integration of long-acting PrEP modalities as they become available. The moderate weight on side effects (17.2%), particularly among younger and online respondents, indicates that proactive counseling about managing transient adverse events could enhance uptake and retention. Conversely, the lower weights for provider type (8.9%) and HIV testing frequency (10.7%) suggest that these attributes may represent secondary considerations for resource allocation. Therefore, prioritizing drug supply consistency and adherence support mechanisms may have a greater impact than expanding provider networks or adjusting testing schedules. This weighted approach supports a data-driven framework for optimizing PrEP delivery by targeting the attributes most influential to PrEP adoption and sustained use among Peruvian SGM.

Our findings contrast with international trends, such as Biello et al.’s research indicating a preference for daily oral PrEP among young MSM in the United States [37]. Nonetheless, our study's in-depth analysis reveals a widespread preference for subcutaneous implant presentations across all participant groups, aligning with prior studies that found increasing interest in less frequent dosing options like injectables and implants among potential ImPrEP participants [26,38,39]. However, the absence of strong preference for once-per-semester dosing suggests that very infrequent intervals may be perceived as less reliable, despite the overall interest in long-acting modalities. This observation underscores that acceptance of ultra–long-acting options may require targeted educational efforts addressing their mechanisms of action and sustained efficacy. Recently, Lenacapavir, a biannual subcutaneous injectable PrEP, has shown high efficacy in HIV prevention, according to preliminary results from the PURPOSE 2 study [19]. While the WHO has recognized Lenacapavir potential, its implementation and scale-up remain challenging in low-resource settings [40]. Moreover, the preference for implant-based PrEP does not contradict the observed favorability towards daily or once-per-trimester dosing. Rather, it reflects varying healthcare needs and priorities, highlighting the crucial need for HIV prevention strategies that are specifically tailored to the unique contexts of different regions, like Peru.

The increased concern about side effects among STI clinic attendees and online groups, particularly among sex workers, calls for a more nuanced understanding of how different groups perceive PrEP. The importance given to the attribute of PrEP presentation and HIV testing frequency among sex workers further emphasizes the need for personalized approaches in PrEP implementation.

This study was not exempt from limitations. The participants in each recruitment group were not randomly selected; therefore, our sample may not be representative of the Peruvian SGM who use PrEP, attend STI clinics, or engage online. Not all possible attributes and their levels could be explored in the experiment. However, we selected those that were most suitable to our social context and that had already been explored in similar scenarios in Latin America [26]. We proposed hypothetical scenarios for the PrEP delivery; however, some attributes were not available. Although long-acting injectable cabotegravir has demonstrated efficacy against HIV infection [16], here is no evidence of its feasible implementation in a Peruvian setting. Similarly, the implant remains a hypothetical preventive approach against HIV; its application in men for other medical indications is still poorly understood [38]. This information gap raises the possibility that the conditions and adherence to these PrEP presentations may be influenced by as unexplored factors. Moreover, there was not an equal proportion of participants who did not answer the DCE questions in each recruitment group. Nearly 20% of individuals recruited via online did not respond to the DCE, while less than 3% of ImPrEP participants and STI clinic attendees recruited did not. The difference may be that those who were recruited at physical sites had the advantage of being assisted by trained interviewers and had more commitment to the study. This difference in response rates may introduce selection bias in the online group and should be considered when comparing preferences across recruitment strategies. However, the online version of the DCE allowed us to obtain the preferences of populations that, for different reasons, cannot approach a health center or institution that provides PrEP. Finally, we can only exclude online participants who were currently ImPrEP participants, but not those who attended STI clinics. Therefore, this may underestimate the differences between both groups.

Our study's timing, conducted before the implementation of Peru's free PrEP policy, provides a baseline against which the impact of this policy can be evaluated. Future research would focus on how these policy changes have influenced PrEP uptake and adherence, especially considering our findings regarding user preferences. Understanding the barriers to accessing PrEP, despite its free provision, is crucial for refining public health strategies and ensuring that they are effectively meeting the needs of key populations.

## Conclusions

Our findings underscore the importance of tailored PrEP delivery methods that consider the unique preferences and challenges of different subgroups within the population. The pronounced concern for side effects and varying preferences for PrEP modalities highlight the need for continuous education and adaptable healthcare services. By addressing these concerns and preferences healthcare providers can improve PrEP acceptability and adherence, ultimately contributing to more effective HIV prevention strategies in Peru and similar settings.

## Supporting information

S1 Table Attributes and levels of PrEP. (PDF)

S2 Questions Survey on preferences for different HIV Pre-Exposure Prophylaxis Schemes. Only available in Spanish.
(PDF)
