# Supplementary material for: Which are the most valued HIV pre-exposure prophylaxis attributes? A discrete choice experiment among sexual and gender minorities in Peru
Source: PLoS One. 2026 Apr 24;21(4):e0346154. doi: 10.1371/journal.pone.0346154 (PMC13108786; doi:10.1371/journal.pone.0346154)
Supplement: S1 Table — (PDF) [file pone.0346154.s001.pdf]

S1 Table. Attributes and levels of PrEP

Attributes	Levels
Presentation	Oral pill
	Injectable (intramuscular)
	Implant (subcutaneous)
Frequency of use	Daily
	Event-Driven
	Monthly
	Once per trimester
	Once per semester
Provider	Public healthcare centers
	Private healthcare centers
	Non-Governmental Organization
Frequency of visits for HIV testing	Once per bimester
	Once per trimester
	Once per semester
Side-effects	None
	Mild and disappear in the first weeks
	Mild and last for weeks
	Moderate
Efficacy	90% - 99% (9 in 10 remain HIV negative)
	80% - 89% (8 in 10 remain HIV negative)
	70% - 79% (7 in 10 remain HIV negative)
