# Supplementary material for: Which are the most valued HIV pre-exposure prophylaxis attributes? A discrete choice experiment among sexual and gender minorities in Peru
Source: PLoS One. 2026 Apr 24;21(4):e0346154. doi: 10.1371/journal.pone.0346154 (PMC13108786; doi:10.1371/journal.pone.0346154)
Supplement: S2 Questions — Only available in Spanish. (PDF) [file pone.0346154.s002.pdf]

S2 Questionnaire - Survey Instrument

Note: The discrete choice experiment (DCE) visual format used in this questionnaire (Questions CBCVF_Random1-12) was adapted from:

Pereira CC de A, Torres TS, Luz PM, Hoagland B, Farias A, Brito JDU, et al. Preferences for pre-exposure prophylaxis (PrEP) among sexual and gender minorities: a discrete choice experiment in Brazil. Lancet Reg Health - Am. 2023;19: 100432. doi:10.1016/j.lana.2023.100432

Published under CC BY 4.0 license (https://creativecommons.org/licenses/by/4.0/).

All other content is original to this study.

Start

Q0a

Consentimiento Informado para Encuesta sobre preferencias hacia diferentes esquemas de Profilaxis pre Exposición a VIH

El equipo de investigación del “Centro de Investigación Interdisciplinaria en Sexualidad, Sida y Sociedad (CIISSS)” de la UPCH desea invitarte a participar en esta investigación sobre el comportamiento, la salud y las preferencias en relación con el uso de la profilaxis pre-exposición (PrEP). Las personas seropositivas para el VIH no son elegibles para el estudio. La encuesta tiene alrededor de 60 preguntas y la mayoría de respuestas tienen opciones para “marcar”. **Tu participación es muy importante** y llevará entre 20-30 minutos.

Por favor, marque la opción que corresponda:

Q0a=1 ◯ He sido contactado porque actualmente participo en ImPrEP

Q0a=2 ◯ He sido contactado porque en mi visita por servicios de asesoramiento, pruebas de ITS o VIH, el personal del establecimiento me invitó a participar.

Q0a=3 ◯ He sido contactado a través de redes sociales (Facebook, Tinder, Grindr, etc.)

Siguiente

q0bb1

A continuación, le brindamos información sobre los procedimientos, el propósito, confidencialidad, riesgos y beneficios del estudio:

1. Propósito del estudio: El objetivo es describir conocimientos y actitudes hacia diferentes regímenes de la Profilaxis Pre Exposición a VIH (PrEP).

2. Procedimientos: Si usted ACEPTA participar responderá algunas preguntas sobre su comportamiento sexual y preferencias en cuanto al cuidado y prevención de VIH. No hay respuestas correctas o incorrectas, solo tu opinión.

3. Confidencialidad: Tus respuestas serán anónimas y no pueden ser rastreadas: no tenemos ninguna información sobre quién eres. En caso seas participante del proyecto ImPrEP, podrás decidir si deseas vincular las respuestas de este cuestionario con la información de ImPrEP. Todo de manera anónima.

4. Riesgos: No hay ningún riesgo por participar en esta encuesta, pero algunas preguntas son sobre temas personales e íntimos; en este sentido, puedes optar por no responder a cualquier pregunta con la que no te sientas cómodo. Asimismo, puedes decidir no participar y/o terminar la encuesta en cualquier momento.

5. Beneficios: Dentro de las preguntas a contestar, podrías aprender más sobre maneras de prevenir VIH. Asimismo, al finalizar la encuesta, te brindamos información sobre lugares para obtener tales servicios. Si actualmente participas en un servicio de prevención de VIH/ITS, el coordinador del estudio te ayudará a navegar a través de los demás servicios disponibles.

6. Dudas y consultas: Si tienes alguna duda o sugerencia, puedes ponerte en contacto con el investigador responsable Dr. Carlos Cáceres al (01)203-3300 o enviarle un correo a carlos.caceres@upch.pe. Si tienes preguntas sobre tus derechos como participante en esta investigación, puede contactar al Comité Institucional de Ética (CIE-UPCH), Dra. Frine Samalvides, presidenta del CIE-UPCH, al teléfono 319 0000 anexo 2271, o al correo electrónico: duict.cieh@oficinas-upch.pe

Para aceptar participar en esta investigación, por favor marque el siguiente casillero.

q0bb1_1 ☐ **ACEPTO responder el cuestionario.** Declaro que he leído y acepto los términos del consentimiento informado.

q0bb2

Para quienes son actualmente participantes de ImPrEP, podríamos vincular tu información entre ambos estudios de manera ANÓNIMA.

q0bb2=1 ○ Soy (o fui) participante de ImPrEP y **ACEPTO** vincular mi información de ambos estudios.

q0bb2=2 ○ Soy (o fui) participante de ImPrEP y **NO ACEPTO** vincular información de ambos estudios.

Atrás | Siguiente

0% 100%

Q0c

A continuación, le brindamos información sobre los procedimientos, el propósito, confidencialidad, riesgos y beneficios del estudio:

1. Propósito del estudio: El objetivo es describir conocimientos y actitudes hacia diferentes regímenes de la Profilaxis Pre Exposición a VIH (PrEP).

2. Procedimientos: Si usted ACEPTA participar responderá algunas preguntas sobre su comportamiento sexual y preferencias en cuanto al cuidado y prevención de VIH. No hay respuestas correctas o incorrectas, solo tu opinión.

3. Confidencialidad: Tus respuestas serán anónimas y no pueden ser rastreadas: no tenemos ninguna información sobre quién eres. En caso seas participante del proyecto ImPrEP, las respuestas del cuestionario podrán ser vinculadas de manera anónima con la información de ImPrEP.

4. Riesgos: No hay ningún riesgo por participar en esta encuesta, pero algunas preguntas son sobre temas delicados; en este sentido, puedes optar por no responder a cualquier pregunta con la que no te sientas cómodo. Asimismo, puedes decidir no participar y/o terminar la encuesta en cualquier momento.

5. Beneficios: Dentro de las preguntas a contestar, podrías aprender más sobre maneras de prevenir VIH. Asimismo, al finalizar la encuesta, te brindamos información sobre lugares para obtener tales servicios. Si actualmente participas en un servicio de prevención de VIH/ITS, el coordinador del estudio te ayudará a navegar a través de los demás servicios disponibles.

6. Dudas y consultas: Si tienes alguna duda o sugerencia, puedes ponerte en contacto con el investigador responsable Dr. Carlos Cáceres al (01)203-3300 o enviarle un correo a carlos.caceres@upch.pe. Si tienes preguntas sobre tus derechos como participante en esta investigación, puede contactar al Comité Institucional de Ética (CIE-UPCH), Dra. Frine Samalvides, presidenta del CIE-UPCH, al teléfono 319 0000 anexo 2271, o escribirle a: Biblioteca Central, Tercer Piso, Av. Honorio Delgado 430, San Martín de Porras, Lima 31, Lima.

Para aceptar participar en esta investigación, **por favor marque el siguiente casillero.**

Q0c_1

☐ **ACEPTO responder el cuestionario. Declaro que he leído y acepto los términos del consentimiento informado.**

Atrás Siguiente

0% 100%

Q0d

Por favor, **ingresa tu "PID"**, con ayuda del coordinador de la sede.

NOTA: Contacta a tu coordinador de ImPrEP para ingresar el PID. ***En caso no sea posible o no recuerdes****, ingresa 14 veces 9, es decir* ***99999999999999***

Atrás Siguiente

0% 100%

Q0e

¿Es la primera vez que contestas este cuestionario?

- Q0e=1 ◯ Sí
- Q0e=2 ◯ No
- Q0e=3 ◯ No sé

Atrás | Siguiente

0% 100%

Q1

¿Cómo se enteró de esta encuesta?

Q1=1 ◯ Facebook

Q1=2 ◯ Grindr

Q1=3 ◯ Hornet

Q1=4 ◯ Instagram

Q1=5 ◯ WhatsApp

Q1=6 ◯ Q1_6_other Otro ____________

Atrás Siguiente

0% 100%

Q2

¿En qué sede ImPrEP o CERITS te encuentras?

Q2=1 ◯ CS Alberto Barton - Callao

Q2=2 ◯ CMI San José - Villa El Salvador

Q2=3 ◯ CMI Tahuantinsuyo bajo - Independencia

Q2=4 ◯ CERITS Tres Compuertas de Caja de Agua - SJL

Q2=5 ◯ CERITS del Hospital La Caleta - Chimbote

Q2=6 ◯ CERITS del Hospital Regional de Ica

Q2=7 ◯ CERITS Hospital Regional Docente de Trujillo

Q2=8 ◯ CERITS San Juan - Iquitos

Q2=9 ◯ UAMP del Hospital Amazónico - Pucallpa

Q2=10 ◯ UAMP del Hospital San Juan de Dios - Pisco

Q2=11 ◯ Hospital Santa Maria del Socorro - Ica

Q2=12 ◯ Hospital Regional de Huacho

Q2=13 ◯ CS Gustavo Lanata - Chorrillos

Q2=14 ◯ CS Villa Primavera - Sullana

Q2=15 ◯ CERITS San Jose - Piura

Q2=16 ◯ CS Madre Teresa de Calcuta

Atrás | Siguiente

0% 100%

Q3

¿Cuántos años tienes?

NOTA: Ingresar en números. [ ]

Q4

¿Cuál fue tu sexo asignado al nacer?

- Q4=1 ○ Hombre
- Q4=2 ○ Mujer
- Q4=3 ○ Intersexual

Q5

¿Con cuál género te identificas actualmente?

- Q5=1 ○ Hombre/Masculino
- Q5=2 ○ Hombre trans
- Q5=3 ○ Mujer
- Q5=4 ○ Mujer trans
- Q5=5 ○ Travesti
- Q5=6 ○ No binario
- Q5=7 ○ Q5_7_other Otro (especifique) [ ]

Q6a

¿Naciste en Perú?

- Q6a=1 ○ Sí
- Q6a=2 ○ No

Atrás Siguiente

0% 100%

Q6b

¿Dónde naciste?

Atrás Siguiente

0% 100%

Q7

¿En cuál departamento/provincia vives actualmente?

Q8

¿Vives en la capital del departamento?

Q8=1 Sí

Q8=2 No

Atrás Siguiente

0% 100%

Q9

¿Cuál fue el nivel o grado de estudios más alto que aprobaste?

Q9=1 ◯ Sin educación

Q9=2 ◯ Inicial/pre-escolar

Q9=3 ◯ Primaria completa

Q9=4 ◯ Secundaria completa

Q9=5 ◯ Superior técnica completa

Q9=6 ◯ Superior universitaria completa

Q9=7 ◯ Postgrado completo

Q10a

¿A cuánto ascienden tus ingresos personales por mes, aproximadamente?

Q10a=1 ◯ No tengo ingresos

Q10a=2 ◯ Menos de 930 nuevos soles

Q10a=3 ◯ De 931 a 1860 nuevos soles

Q10a=4 ◯ De 1861 a 2790 nuevos soles

Q10a=5 ◯ De 2791 a 3720 nuevos soles

Q10a=6 ◯ De 3721 a 5580 nuevos soles

Q10a=7 ◯ De 5581 a 9300 nuevos soles

Q10a=8 ◯ De 9301 nuevos soles a más

Q10a=9 ◯ No quiero responder

q10b

Considerando todos los ingresos de tu familia o personas que viven contigo, ¿a cuánto ascienden los ingresos de tu casa por mes, aproximadamente?

NOTA: Considera solo aquellos familiares o personas que contribuyen a los gastos de casa.

q10b=1
No tengo ingresos

q10b=2
Menos de 930 nuevos soles

q10b=3
De 931 a 1860 nuevos soles

q10b=4
De 1861 a 2790 nuevos soles

q10b=5
De 2791 a 3720 nuevos soles

q10b=6
De 3721 a 5580 nuevos soles

q10b=7
De 5581 a 9300 nuevos soles

q10b=8
De 9301 nuevos soles a más

q10b=9
No quiero responder

Q11

Por sus antepasados y de acuerdo a sus costumbres, ¿ud. se considera?

Q11=3
Nativo o Indígena de la Amazonía

Q11=6
Blanco

Q11=8
Asiático

Q11=7
Mestizo

Q11=1
Quechua

Q11=5
Afroperuano/Negro/Moreno/Mulato

Q11=2
Aymara

Q11=4
Perteneciente o parte de otro Pueblo indígena u originario

Q11=9
Q11_9_other
Otro (especifique)

Q12

¿Tu atracción sexual es preferentemente hacia?

Q12=1
Hombres

Q12=2
Mujeres

Q12=3
Hombres/Mujeres

Q12=4
No quiero responder

Q12=5
Q12_5_other
Otro (especifique)

Q13
Actualmente, ¿tienes pareja(s) fija(s) estable(s)?

Q13=1
Sí

Q13=2
No

Atrás   Siguiente

0% 100%

Q13b

¿Tu(s) pareja(s) fija(s) viven con VIH?

Q13b=1 Sí

Q13b=2 No

Q13b=3 No sé

Atrás Siguiente

0% 100%

Q22

¿Cuándo fue la última vez que te hiciste una prueba para el VIH?

Q22=1 ○ En los últimos 3 meses

Q22=2 ○ En los últimos 6 meses

Q22=3 ○ En el último año

Q22=4 ○ Fue hace más de un año

Q22=5 ○ Nunca

Q22=6 ○ No sé / No quiero responder

Atrás | Siguiente

0% 100%

Q23

¿Cuál es la razón principal por la que nunca te has hecho una prueba para el VIH?

Q23=1 No estoy en riesgo

Q23=2 Me parece poco práctico o incómodo ir a un servicio de salud

Q23=3 Tengo vergüenza de hacerme la prueba

Q23=4 Tengo miedo de resultar positivo

Q23=5 Me da pereza hacerlo

Q23=6 No quiero responder

Atrás Siguiente

0% 100%

q26d

¿Alguna vez has oído hablar de Indetectable = Intransmitable (I=I)?

q26d=1 Sí

q26d=2 No

q26d=3 No quiero responder

Atrás Siguiente

0% 100%

q26e

El concepto de Indetectable=Intransmisible (I=I) significa que las personas con VIH y una carga viral indetectable no transmiten la infección por relaciones sexuales sin protección.

¿Qué tan correcta consideras esta definición?

q26e=1 Totalmente correcta

q26e=2 Un poco correcta

q26e=3 Un poco incorrecta

q26e=4 Totalmente incorrecta

q26e=5 No sé qué significa ser "indetectable"

Atrás Siguiente

0% 100%

Q24

¿Has tenido algún resultado positivo para el VIH?

Q24=1 ◯ Sí

Q24=2 ◯ No

Q24=3 ◯ No quiero responder

Atrás Siguiente

0% 100%

Q25

¿Por cuánto tiempo has vivido con el VIH?

Q25=1 ◯ Menos de seis meses

Q25=2 ◯ 6 meses a menos de 1 año

Q25=3 ◯ 1-5 años

Q25=4 ◯ 6-10 años

Q25=5 ◯ Más de 10 años

Q25=6 ◯ No quiero responder

Q26

¿Está actualmente tomando terapia antirretroviral?

Q26=1 ◯ Sí

Q26=2 ◯ No

Q26=3 ◯ No quiero responder

Atrás | Siguiente

0% 100%

q26a

¿Por qué no estás tomando terapia antirretroviral? (Marque todo lo que corresponda)

q26a_1 ☐ No creo que sea necesario porque mi salud está bien

q26a_2 ☐ Mi médico me dijo que soy un controlador élite de VIH, por lo que no necesito tratamiento

q26a_3 ☐ No tengo acceso al tratamiento

q26a_4 ☐ El conteo de mi CD4 es bueno, así que no necesito tratamiento.

q26a_5 ☐ El gasto para estar en tratamiento es demasiado alto para mi (transporte, pagos, etc.)

q26a_6 ☐ Tengo problemas para acceder a los servicios de VIH (distancia o tiempo que pasé en el centro)

q26a_7 ☐ Me preocupa el estigma / no quiero ser identificado como persona viviendo con VIH

q26a_8 ☐ Me preocupa cómo seré tratado(a) por los profesionales de la salud

q26a_9 ☐ Estoy usando medicina tradicional para tratar el VIH

q26a_10 ☐ Me preocupan los efectos secundarios del tratamiento anti-retroviral

q26a_11 ☐ No sé dónde puedo obtener tratamiento para el VIH

q26a_12 ☐ No tengo seguro de salud

q26a_13 ☐ No quiero responder

q26a_14 ☐ q26a_14_other Otro (especifique) ________

Atrás Siguiente

0% 100%

q26b
¿Cuándo fue tu última prueba de carga viral?

- q26b=1 ◯ Menos de tres meses
- q26b=2 ◯ Menos de seis meses
- q26b=3 ◯ Menos de un año
- q26b=4 ◯ Más de un año
- q26b=5 ◯ No recuerdo
- q26b=6 ◯ Nunca he hecho la prueba de carga viral
- q26b=7 ◯ No quiero responder

q26c
¿En tu última visita de salud tuviste un resultado de carga viral indetectable (ej. infección de VIH controlada) ?

- q26c=1 ◯ Sí
- q26c=2 ◯ No
- q26c=3 ◯ No quiero responder

Atrás Siguiente

0% 100%

Q36

¿Has escuchado de PrEP (profilaxis pre-exposición)?

Q36=1 Sí

Q36=2 No

Atrás Siguiente

0% 100%

Q37

Profilaxis pre-exposición, también llamada PrEP, es el uso preventivo de medicamentos antirretrovirales para prevenir la infección de VIH en personas que no tienen VIH. Hay evidencia de múltiples estudios que muestran que la PrEP funciona, si se toma correctamente. La PrEP ahora es recomendado por la OMS para personas en riesgo de adquirir VIH. Para tomar PrEP se requiere visitar a un médico cada tres meses y hacerse pruebas de VIH/ITS.

¿Actualmente estás tomando PrEP?

- Q37=1 No
- Q37=2 Sí, a través de un proyecto (ej. ImPrEP)
- Q37=3 Sí, tomo PrEP en un servicio privado y yo me la compro
- Q37=4 Sí, tomo PrEP porque mi(s) amigo(s) o pareja(s) me la dan.

Atrás Siguiente

0% 100%

Q38

¿Has tomado PrEP alguna vez en tu vida?

- Q38=1 ◯ Nunca he tomado PrEP
- Q38=2 ◯ Sí, pero la dejé porque mi riesgo de VIH disminuyó
- Q38=3 ◯ Sí, pero la dejé porque ya no quería tomar PrEP
- Q38=4 ◯ Sí, pero la dejé porque el proyecto en el que estaba terminó
- Q38=5 ◯ Sí, pero la dejé por otro motivo (especifique) Q38_5_other [ ]

Atrás | Siguiente

0% 100%

Q39

¿Cuánto tiempo tomaste o estás tomando PrEP?

Q39=1 ◯ Menos de 3 meses

Q39=2 ◯ De 3 - 6 meses

Q39=3 ◯ Más de 6 meses

Atrás | Siguiente

0% 100%

Q45

¿Qué tan probable sería que estés dispuesto(a) a usar PrEP oral diario para prevenir infección por VIH?

Q45=1 ◯ Muy poco probable

Q45=2 ◯ Poco probable

Q45=3 ◯ Algo probable

Q45=4 ◯ Muy probable

Atrás Siguiente

0% 100%

Q45b

Considerando tu ingreso mensual y gastos, ¿cuánto estarías dispuesto(a) a pagar por **un mes de medicamento** de la PrEP de toma diaria? (Anota la cantidad en **SOLES**. Escribe "0" si no estarías dispuesto a pagar nada)

S/ [ ]

Q46

¿Cuán probable sería que usaras PrEP para prevenir el VIH si te lo ofrecieran gratuitamente en los servicios del Ministerio de Salud en tu zona o ciudad?

- Q46=1 ○ Muy poco probable
- Q46=2 ○ Poco probable
- Q46=3 ○ Algo probable
- Q46=4 ○ Muy probable

Atrás Siguiente

0% 100%

Q40

En una escala de "Nada importante" a "Muy importante", por favor señala la importancia que tienen para ti las siguientes oraciones en tu decisión de tomar la PrEP:

	Nada importante	Algo importante	Importante	Muy importante
Temor a que la PreP pudiera tener efectos secundarios inmediatos en mi salud.	Q40_r1=1 ◯	Q40_r1=2 ◯	Q40_r1=3 ◯	Q40_r1=4 ◯
Temor a que la PrEP pudiera tener efectos secundarios a largo plazo en mi salud	Q40_r2=1 ◯	Q40_r2=2 ◯	Q40_r2=3 ◯	Q40_r2=4 ◯
Temor a que los medicamentos para tratar el VIH no funcionen porque estuve tomando PrEP, si adquiriera la infección	Q40_r3=1 ◯	Q40_r3=2 ◯	Q40_r3=3 ◯	Q40_r3=4 ◯
Temor a que la PrEP no proteja al 100% para prevenir el VIH	Q40_r4=1 ◯	Q40_r4=2 ◯	Q40_r4=3 ◯	Q40_r4=4 ◯

Atrás Siguiente

0% 100%

Q41

En una escala de "nada importante" a "muy importante", por favor indica que tan importante sería la siguiente preocupación en tu decisión de tomar PrEP:

	Nada importante	Algo importante	Importante	Muy importante	No uso terapia hormonal
Temor a que la PrEP pudiera interferir con mi terapia hormonal	Q41_r1=1 ○	Q41_r1=2 ○	Q41_r1=3 ○	Q41_r1=4 ○	Q41_r1=5 ○

Atrás Siguiente

0% 100%

Q42

¿Alguna vez ha escuchado sobre "PrEP por evento", "2-1-1 PrEP" o "PrEP a demanda"?

Q42=1 Sí

Q42=2 No

Atrás Siguiente

0% 100%

Q43

La Organización Mundial de la Salud ha aprobado la "PrEP por evento" o "ED-PrEP" para hombres que tienen sexo con hombres (HSH). Según la OMS: “La PrEP por evento consiste en el uso de una doble dosis (2 pastillas) de PrEP entre 2 y 24 horas antes del sexo; luego, una tercera pastilla 24 horas después de las dos primeras pastillas, y una cuarta pastilla 24 horas después de la tercera pastilla". La recomendación es para HSH que tienen relaciones sexuales predecibles (con al menos 2 horas de anticipación) y que tienen relaciones sexuales menos de 2 veces por semana. Hasta ahora, no hay suficiente evidencia científica para recomendar "PrEP por evento" para personas transgénero.

	Muy poco probable	Poco probable	Algo probable	Muy probable
¿Cuán probable seria que uses el método "PrEP por evento" para prevenir la infección por VIH?	Q43_r1=1 ○	Q43_r1=2 ○	Q43_r1=3 ○	Q43_r1=4 ○

Atrás Siguiente

0% 100%

Q44a

Considerando tu disposición a usar la "PrEP por evento", ¿qué tan de acuerdo estás con las siguientes afirmaciones?

	Totalmente en desacuerdo	Algo en desacuerdo	Algo de acuerdo	Totalmente de acuerdo
Puedo anticipar muy bien cuándo tendré sexo	Q44a_r1=1 ○	Q44a_r1=2 ○	Q44a_r1=3 ○	Q44a_r1=4 ○
Puedo posponer el sexo por al menos dos horas	Q44a_r2=1 ○	Q44a_r2=2 ○	Q44a_r2=3 ○	Q44a_r2=4 ○
La PrEP diaria parece dañina para mi cuerpo	Q44a_r3=1 ○	Q44a_r3=2 ○	Q44a_r3=3 ○	Q44a_r3=4 ○
Me gusta tomar pastillas solo cuando las necesito	Q44a_r4=1 ○	Q44a_r4=2 ○	Q44a_r4=3 ○	Q44a_r4=4 ○
Tengo miedo de los efectos adversos a largo plazo si tomo PrEP diariamente	Q44a_r5=1 ○	Q44a_r5=2 ○	Q44a_r5=3 ○	Q44a_r5=4 ○
Me resulta difícil recordar tomar 1 pastilla al día	Q44a_r6=1 ○	Q44a_r6=2 ○	Q44a_r6=3 ○	Q44a_r6=4 ○
No me gusta tomar pastillas diariamente	Q44a_r7=1 ○	Q44a_r7=2 ○	Q44a_r7=3 ○	Q44a_r7=4 ○
Tomar "PrEP por evento" sería más conveniente para mí	Q44a_r8=1 ○	Q44a_r8=2 ○	Q44a_r8=3 ○	Q44a_r8=4 ○
PrEP por evento es tan efectivo como la PrEP diaria	Q44a_r9=1 ○	Q44a_r9=2 ○	Q44a_r9=3 ○	Q44a_r9=4 ○
Tengo un riesgo pequeño / moderado de contraer el VIH sin usar PrEP	Q44a_r10=1 ○	Q44a_r10=2 ○	Q44a_r10=3 ○	Q44a_r10=4 ○
Tengo relaciones sexuales, en promedio, menos de 2 veces por semana	Q44a_r11=1 ○	Q44a_r11=2 ○	Q44a_r11=3 ○	Q44a_r11=4 ○

Atrás | Siguiente

0% 100%

Q44b

Considerando la poca probabilidad de que usaras la "PrEP por evento", ¿qué tan de acuerdo estás con las siguientes afirmaciones?

	Totalmente en desacuerdo	Algo en desacuerdo	Algo de acuerdo	Totalmente de acuerdo
No puedo planear cuándo tendré sexo	Q44b_r1=1 ◯	Q44b_r1=2 ◯	Q44b_r1=3 ◯	Q44b_r1=4 ◯
La PrEP a demanda es tan efectiva como la PrEP diaria	Q44b_r2=1 ◯	Q44b_r2=2 ◯	Q44b_r2=3 ◯	Q44b_r2=4 ◯
Me sentiría ansioso acerca de mi riesgo de VIH si tomara PrEP a demanda, en lugar de PrEP diaria	Q44b_r3=1 ◯	Q44b_r3=2 ◯	Q44b_r3=3 ◯	Q44b_r3=4 ◯
Me sentiría más cómodo tomando PrEP diaria que PrEP a demanda	Q44b_r4=1 ◯	Q44b_r4=2 ◯	Q44b_r4=3 ◯	Q44b_r4=4 ◯
PrEP a demanda es un régimen difícil, prefiero algo más regular	Q44b_r5=1 ◯	Q44b_r5=2 ◯	Q44b_r5=3 ◯	Q44b_r5=4 ◯
En promedio, tengo sexo más de 2 veces a la semana	Q44b_r6=1 ◯	Q44b_r6=2 ◯	Q44b_r6=3 ◯	Q44b_r6=4 ◯
No puedo posponer el sexo no planeado más de 2 horas	Q44b_r7=1 ◯	Q44b_r7=2 ◯	Q44b_r7=3 ◯	Q44b_r7=4 ◯

Atrás Siguiente

0% 100%

Q47

En el último mes, en promedio, ¿cuántos días por semana has tomado tus pastillas de PrEP?

- Q47=1 ○ 1 dia
- Q47=2 ○ 2 días
- Q47=3 ○ 3 días
- Q47=4 ○ 4 días
- Q47=5 ○ 5 días
- Q47=6 ○ 6 días
- Q47=7 ○ Todos los días

Atrás Siguiente

0% 100%

Q48

¿Cuáles fueron las razones por la que no tomaste PrEP todos los días?: (marque todo lo que corresponda)

Q48=1 ◯ Olvidé tomarlo

Q48=2 ◯ Estaba fuera de casa/de viaje

Q48=3 ◯ Se me acabaron las pastillas

Q48=4 ◯ Tuve efectos secundarios

Q48=5 ◯ No pude ir a mi cita de seguimiento por la COVID-19

Q48=6 ◯ Q48_6_other Otro (especifique) ____________

Atrás | Siguiente

0% 100%

Q49

¿Cómo estás tomando la PrEP actualmente?

Q49=1 ○ Tomo una pastilla al día

Q49=2 ○ Cuando me siento en riesgo, tomo 2 pastillas entre 2-24 horas antes de tener relaciones sexuales, 1 pastilla 24 horas después de las dos primeras pastillas, y 1 pastilla 48 horas después de las dos primeras pastillas

Q49=3 ○ Solo la tomo cuando me siento en riesgo

Q49=4 ○ Q49_4_other Otro [ ]

Atrás | Siguiente

0% ▬▬▬▬ 100%

Q50

Considerando tus prácticas sexuales actuales, en tu opinión, ¿cuál sería tu riesgo de adquirir VIH en los próximos 12 meses **si no estuvieras tomando la PrEP** ?

Q50=1 Sin riesgo

Q50=2 Riesgo bajo

Q50=3 50% de riesgo

Q50=4 Alto riesgo

Q50=5 100%

Q50=6 No quiero responder

Atrás Siguiente

0% 100%

Q51

¿Cuál de las siguientes opciones te ayudarían a recordar la toma de PrEP? (Marca todas las que apliquen)

- Q51_1 ☐ SMS - Mensaje de texto al celular
- Q51_2 ☐ Mensajes por WhatsApp
- Q51_3 ☐ Mensajes por redes sociales (Facebook, Twitter, Instagram, etc.)
- Q51_4 ☐ Visitas regulares al centro de salud
- Q51_5 ☐ Recordatorios de un amigo(a) o miembro de la familia
- Q51_6 ☐ Una alerta en el calendario de mi celular / poner una alarma
- Q51_7 ☐ Usar un pastillero rellenado cada semana
- Q51_8 ☐ Ser parte de un grupo de apoyo para usar PrEP como está prescrita
- Q51_9 ☐ Ninguna de las opciones anteriores
- Q51_10 ☐ Q51_10_other Otra (especifique) ______

Q52

Si estuvieran disponibles las siguientes modalidades de PrEP, ¿qué tan probable sería que las usaras?

	Muy poco probable	Poco probable	Neutro	Probable	Muy probable
PrEP inyectable (1 inyección en la nalga cada dos meses)	Q52_r1=1 ○	Q52_r1=2 ○	Q52_r1=3 ○	Q52_r1=4 ○	Q52_r1=5 ○
PrEP tópico diario (aplicación de gel en la zona anal todos los días)	Q52_r2=1 ○	Q52_r2=2 ○	Q52_r2=3 ○	Q52_r2=4 ○	Q52_r2=5 ○
PrEP tópico por evento (aplicación de gel en la zona anal 12 horas antes y después del sexo)	Q52_r3=1 ○	Q52_r3=2 ○	Q52_r3=3 ○	Q52_r3=4 ○	Q52_r3=5 ○
PrEP oral mensual (1 pastilla 1 vez por mes)	Q52_r4=1 ○	Q52_r4=2 ○	Q52_r4=3 ○	Q52_r4=4 ○	Q52_r4=5 ○
Parche de PrEP (un parche en la piel que se cambiaría periódicamente)	Q52_r5=1 ○	Q52_r5=2 ○	Q52_r5=3 ○	Q52_r5=4 ○	Q52_r5=5 ○
PrEP implante (pequeño implante de 3cm debajo de la piel)	Q52_r6=1 ○	Q52_r6=2 ○	Q52_r6=3 ○	Q52_r6=4 ○	Q52_r6=5 ○
Inyección intravenosa de anticuerpos monoclonales (suero de anticuerpos contra el VIH, puesto en la vena)	Q52_r7=1 ○	Q52_r7=2 ○	Q52_r7=3 ○	Q52_r7=4 ○	Q52_r7=5 ○

Inyección sub-dérmica de anticuerpos monoclonales (inyección debajo de la piel con anticuerpos contra el VIH)	Q52_r8=1 ◯	Q52_r8=2 ◯	Q52_r8=3 ◯	Q52_r8=4 ◯	Q52_r8=5 ◯
Vacuna contra el VIH	Q52_r9=1 ◯	Q52_r9=2 ◯	Q52_r9=3 ◯	Q52_r9=4 ◯	Q52_r9=5 ◯

Atrás Siguiente

0% 100%

CBCVF_Random1

Imagina que puedes acceder a diferentes opciones de **PrEP**. A continuación te mostramos 12 preguntas donde debes escoger entre **2 opciones potenciales**. En cada pregunta **elige cuál te interesaría más**.

NOTA: El botón para escoger está en la parte inferior. Luego de escoger tu opción preferida, presiona el botón "Siguiente".

(1 of 12)

	Opción A	Opción B
Tipo de PrEP	**Inyectable** 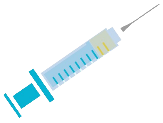	**Oral** 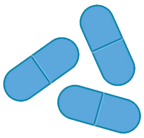
Frecuencia de uso	**1 vez al mes** 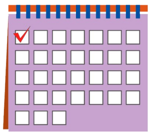	**PrEP por evento (2-1-1)** 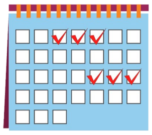
Proveedor de PrEP	**Sector Privado<br>Con costo<br>Menor espera** 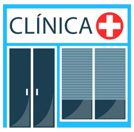	**ONG<br>Costo mínimo<br>Menor espera** 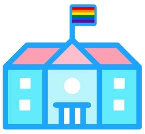
Frecuencia de prueba de VIH	**Prueba de VIH cada 3 meses** 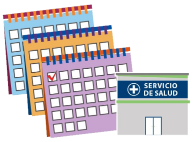	**Prueba de VIH cada 3 meses** 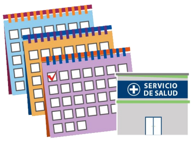
Efectos secundarios	**Leves y desaparecen en las primeras semanas** 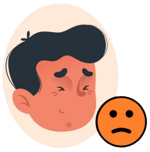	**Sin efectos secundarios** 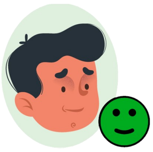
Nivel de eficacia	**El 80% que usa PrEP sigue siendo VIH negativo** 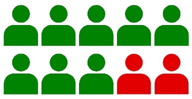	**El 90% que usa PrEP sigue siendo VIH negativo** 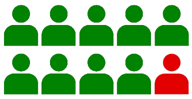

CBCVF_Random1

Elijo esta opción

CBCVF_Random1

Elijo esta opción

Atrás

Siguiente

0% 100%

CBCVF_Random2

Imagina que puedes acceder a diferentes opciones de **PrEP**. A continuación te mostramos 12 preguntas donde debes escoger entre **2 opciones potenciales**. En cada pregunta **elige cuál te interesaría más**.

NOTA: El botón para escoger está en la parte inferior. Luego de escoger tu opción preferida, presiona el botón "Siguiente".

(2 of 12)

	Opción A	Opción B
Tipo de PrEP	Oral 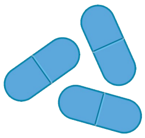	Implante (subcutáneo) 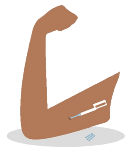
Frecuencia de uso	Diario 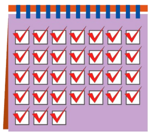	1 vez cada 6 meses 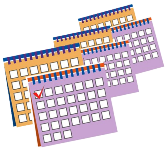
Proveedor de PrEP	Sector Público Gratuito Mayor espera 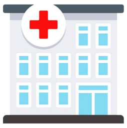	Sector Público Gratuito Mayor espera 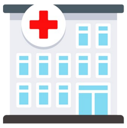
Frecuencia de prueba de VIH	Prueba de VIH cada 3 meses 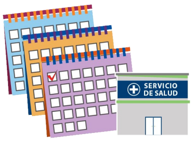	Prueba de VIH cada 6 meses 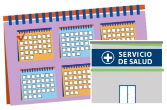
Efectos secundarios	Leves y desaparecen en las primeras semanas 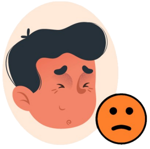	Leves y permanecen durante semanas 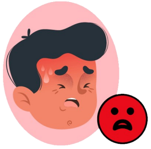
Nivel de eficacia	El 70% que usa PrEP sigue siendo VIH negativo 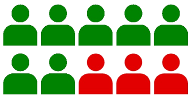	El 90% que usa PrEP sigue siendo VIH negativo 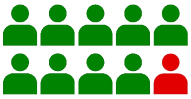

CBCVF_Random2

Elijo esta opción

CBCVF_Random2

Elijo esta opción

Atrás Siguiente

0% 100%

CBCVF_Random3

Imagina que puedes acceder a diferentes opciones de **PrEP**. A continuación te mostramos 12 preguntas donde debes escoger entre **2 opciones potenciales**. En cada pregunta **elige cuál te interesaría más**.

NOTA: El botón para escoger está en la parte inferior. Luego de escoger tu opción preferida, presiona el botón "Siguiente".

(3 of 12)

	Opción A	Opción B
Tipo de PrEP	**Inyectable** 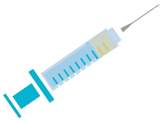	**Implante** (subcutáneo) 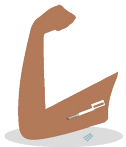
Frecuencia de uso	**1 vez cada 2 - 3 meses** 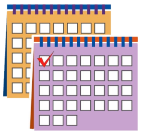	**1 vez cada 6 meses** 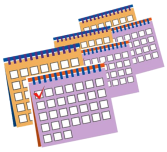
Proveedor de PrEP	**ONG**<br>**Costo mínimo**<br>**Menor espera** 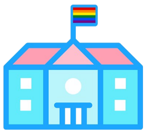	**Sector Privado**<br>**Con costo**<br>**Menor espera** 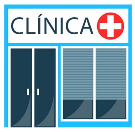
Frecuencia de prueba de VIH	**Prueba de VIH cada 3 meses** 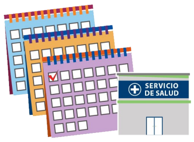	**Prueba de VIH cada 6 meses** 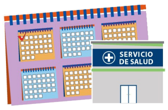
Efectos secundarios	**Leves y permanecen durante semanas** 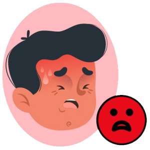	**Sin efectos secundarios** 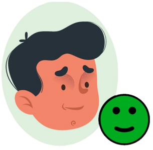
Nivel de eficacia	**El 70% que usa PrEP sigue siendo VIH negativo** 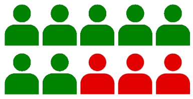	**El 80% que usa PrEP sigue siendo VIH negativo** 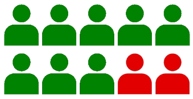

CBCVF_Random3

Elijo esta opción

CBCVF_Random3

Elijo esta opción

Atrás Siguiente

0% 100%

CBCVF_Random4

Imagina que puedes acceder a diferentes opciones de **PrEP**. A continuación te mostramos 12 preguntas donde debes escoger entre **2 opciones potenciales**. En cada pregunta **elige cuál te interesaría más**.

NOTA: El botón para escoger está en la parte inferior. Luego de escoger tu opción preferida, presiona el botón "Siguiente".

(4 of 12)

	Opción A	Opción B
Tipo de PrEP	**Implante** (subcutáneo) 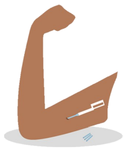	**Inyectable** 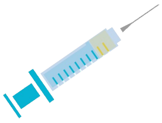
Frecuencia de uso	**1 vez cada 6 meses** 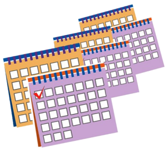	**1 vez al mes** 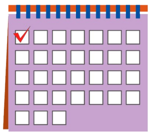
Proveedor de PrEP	**Sector Público**<br>**Gratuito**<br>**Mayor espera** 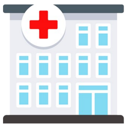	**ONG**<br>**Costo mínimo**<br>**Menor espera** 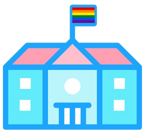
Frecuencia de prueba de VIH	**Prueba de VIH cada 6 meses** 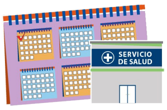	**Prueba de VIH cada 3 meses** 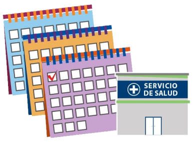
Efectos secundarios	**Sin efectos secundarios** 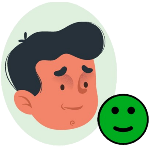	**Leves y permanecen durante semanas** 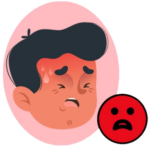
Nivel de eficacia	**El 80% que usa PrEP sigue siendo VIH negativo** 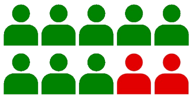	**El 90% que usa PrEP sigue siendo VIH negativo** 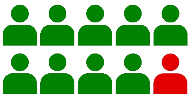

CBCVF_Random4

Elijo esta opción

CBCVF_Random4

Elijo esta opción

Atrás Siguiente

0% 100%

CBCVF_Random5

Imagina que puedes acceder a diferentes opciones de **PrEP**. A continuación te mostramos 12 preguntas donde debes escoger entre **2 opciones potenciales**. En cada pregunta **elige cuál te interesaría más**.

NOTA: El botón para escoger está en la parte inferior. Luego de escoger tu opción preferida, presiona el botón "Siguiente".

(5 of 12)

	Opción A	Opción B
Tipo de PrEP	**Oral** 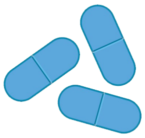	**Implante** (subcutáneo) 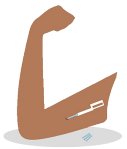
Frecuencia de uso	**Diario** 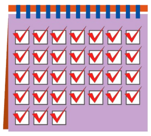	**1 vez cada 6 meses** 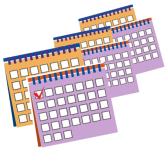
Proveedor de PrEP	**Sector Privado Con costo Menor espera** 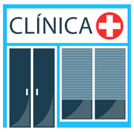	**Sector Público Gratuito Mayor espera** 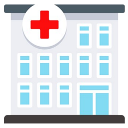
Frecuencia de prueba de VIH	**Prueba de VIH cada 3 meses** 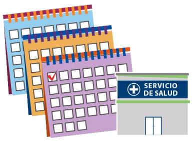	**Prueba de VIH cada 6 meses** 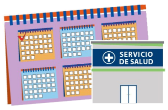
Efectos secundarios	**Leves y desaparecen en las primeras semanas** 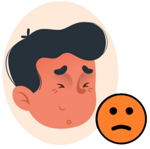	**Leves y permanecen durante semanas** 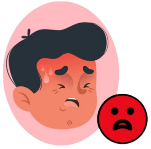
Nivel de eficacia	**El 70% que usa PrEP sigue siendo VIH negativo** 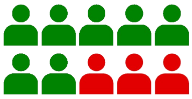	**El 80% que usa PrEP sigue siendo VIH negativo** 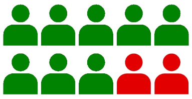

CBCVF_Random5

Elijo esta opción

CBCVF_Random5

Elijo esta opción

Atrás Siguiente

0% 100%

CBCVF_Random6

Imagina que puedes acceder a diferentes opciones de **PrEP**. A continuación te mostramos 12 preguntas donde debes escoger entre **2 opciones potenciales**. En cada pregunta **elige cuál te interesaría más**.

NOTA: El botón para escoger está en la parte inferior. Luego de escoger tu opción preferida, presiona el botón "Siguiente".

(6 of 12)

	Opción A	Opción B
Tipo de PrEP	**Inyectable** 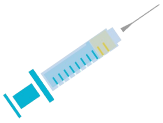	**Oral** 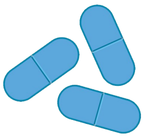
Frecuencia de uso	**1 vez cada 2 - 3 meses** 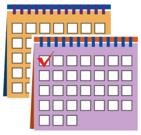	**PrEP por evento (2-1-1)** 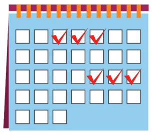
Proveedor de PrEP	**ONG**<br>**Costo mínimo**<br>**Menor espera** 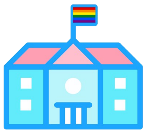	**ONG**<br>**Costo mínimo**<br>**Menor espera** 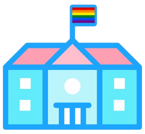
Frecuencia de prueba de VIH	**Prueba de VIH cada 3 meses** 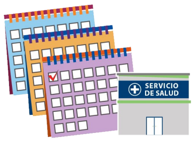	**Prueba de VIH cada 3 meses** 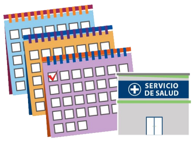
Efectos secundarios	**Sin efectos secundarios** 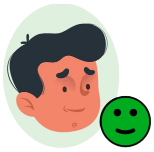	**Leves y desaparecen en las primeras semanas** 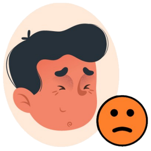
Nivel de eficacia	**El 70% que usa PrEP sigue siendo VIH negativo** 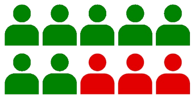	**El 80% que usa PrEP sigue siendo VIH negativo** 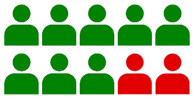

CBCVF_Random6

Elijo esta opción

CBCVF_Random6

Elijo esta opción

Atrás Siguiente

0% 100%

CBCVF_Random7

Imagina que puedes acceder a diferentes opciones de **PrEP**. A continuación te mostramos 12 preguntas donde debes escoger entre **2 opciones potenciales**. En cada pregunta **elige cuál te interesaría más**.

NOTA: El botón para escoger está en la parte inferior. Luego de escoger tu opción preferida, presiona el botón "Siguiente".

(7 of 12)

	Opción A	Opción B
Tipo de PrEP	**Implante** (subcutáneo)<br>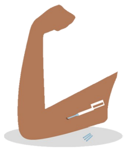	**Inyectable**<br>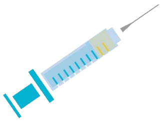
Frecuencia de uso	**1 vez cada 6 meses**<br>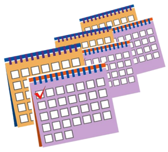	**1 vez al mes**<br>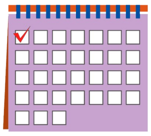
Proveedor de PrEP	**Sector Privado**<br>**Con costo**<br>**Menor espera**<br>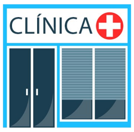	**Sector Público**<br>**Gratuito**<br>**Mayor espera**<br>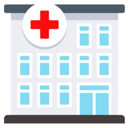
Frecuencia de prueba de VIH	**Prueba de VIH cada 6 meses**<br>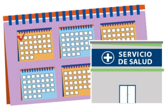	**Prueba de VIH cada 3 meses**<br>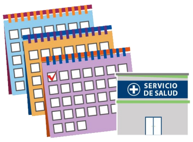
Efectos secundarios	**Leves y permanecen durante semanas**<br>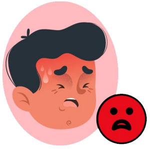	**Sin efectos secundarios**<br>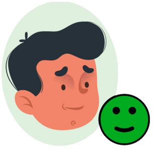
Nivel de eficacia	**El 70% que usa PrEP sigue siendo VIH negativo**<br>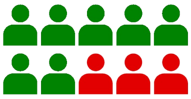	**El 80% que usa PrEP sigue siendo VIH negativo**<br>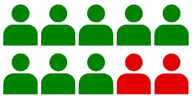

CBCVF_Random7

Elijo esta opción

CBCVF_Random7

Elijo esta opción

Atrás Siguiente

0% 100%

CBCVF_Random8

Imagina que puedes acceder a diferentes opciones de **PrEP**. A continuación te mostramos 12 preguntas donde debes escoger entre **2 opciones potenciales**. En cada pregunta **elige cuál te interesaría más**.

NOTA: El botón para escoger está en la parte inferior. Luego de escoger tu opción preferida, presiona el botón "Siguiente".

(8 of 12)

	Opción A	Opción B
Tipo de PrEP	**Implante** (subcutáneo) 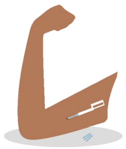	**Oral** 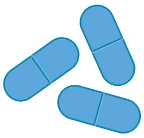
Frecuencia de uso	**1 vez cada 6 meses** 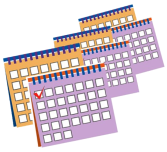	**Diario** 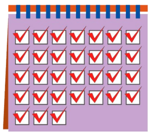
Proveedor de PrEP	**ONG**<br>**Costo mínimo**<br>**Menor espera** 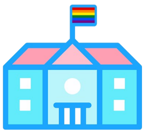	**Sector Público**<br>**Gratuito**<br>**Mayor espera** 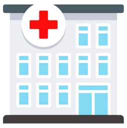
Frecuencia de prueba de VIH	**Prueba de VIH cada 6 meses** 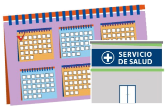	**Prueba de VIH cada 3 meses** 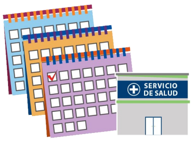
Efectos secundarios	**Leves y desaparecen en las primeras semanas** 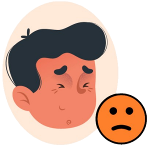	**Leves y permanecen durante semanas** 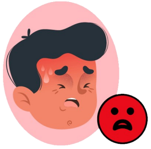
Nivel de eficacia	**El 90% que usa PrEP sigue siendo VIH negativo** 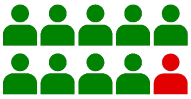	**El 90% que usa PrEP sigue siendo VIH negativo** 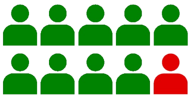

CBCVF_Random8

Elijo esta opción

CBCVF_Random8

Elijo esta opción

Atrás Siguiente

0% 100%

CBCVF_Random9

Imagina que puedes acceder a diferentes opciones de **PrEP**. A continuación te mostramos 12 preguntas donde debes escoger entre **2 opciones potenciales**. En cada pregunta **elige cuál te interesaría más**.

NOTA: El botón para escoger está en la parte inferior. Luego de escoger tu opción preferida, presiona el botón "Siguiente".

(9 of 12)

	Opción A	Opción B
Tipo de PrEP	**Oral** 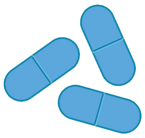	**Oral** 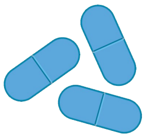
Frecuencia de uso	**Diario** 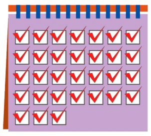	**PrEP por evento (2-1-1)** 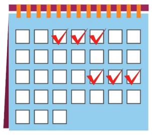
Proveedor de PrEP	**Sector Público**<br>**Gratuito**<br>**Mayor espera** 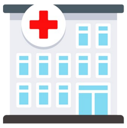	**Sector Privado**<br>**Con costo**<br>**Menor espera** 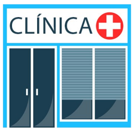
Frecuencia de prueba de VIH	**Prueba de VIH cada 3 meses** 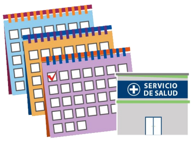	**Prueba de VIH cada 3 meses** 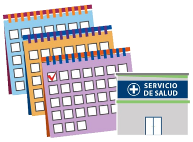
Efectos secundarios	**Sin efectos secundarios** 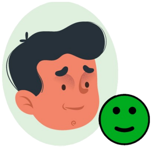	**Leves y desaparecen en las primeras semanas** 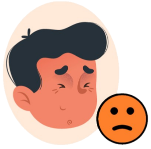
Nivel de eficacia	**El 90% que usa PrEP sigue siendo VIH negativo** 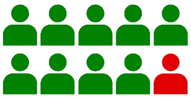	**El 80% que usa PrEP sigue siendo VIH negativo** 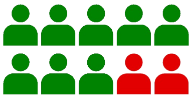

CBCVF_Random9

Elijo esta opción

CBCVF_Random9

Elijo esta opción

Atrás Siguiente

0% 100%

CBCVF_Random10

Imagina que puedes acceder a diferentes opciones de **PrEP**. A continuación te mostramos 12 preguntas donde debes escoger entre **2 opciones potenciales**. En cada pregunta **elige cuál te interesaría más**.

NOTA: El botón para escoger está en la parte inferior. Luego de escoger tu opción preferida, presiona el botón "Siguiente".

(10 of 12)

	Opción A	Opción B
Tipo de PrEP	**Implante** (subcutáneo) 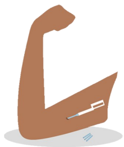	**Inyectable** 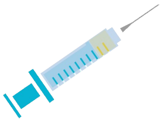
Frecuencia de uso	**1 vez cada 6 meses** 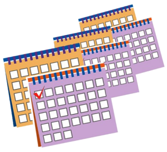	**1 vez cada 2 - 3 meses** 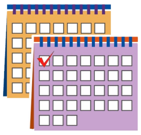
Proveedor de PrEP	Sector Público<br>Gratuito<br>Mayor espera 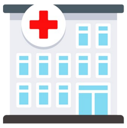	Sector Privado<br>Con costo<br>Menor espera 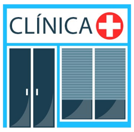
Frecuencia de prueba de VIH	**Prueba de VIH cada 6 meses** 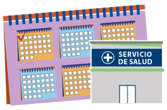	**Prueba de VIH cada 3 meses** 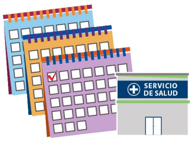
Efectos secundarios	Leves y permanecen durante semanas 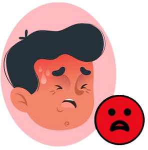	Leves y desaparecen en las primeras semanas 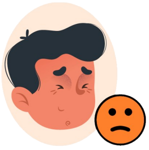
Nivel de eficacia	El 70% que usa PrEP sigue siendo VIH negativo 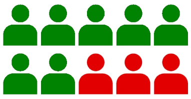	El 70% que usa PrEP sigue siendo VIH negativo 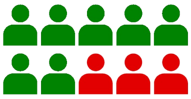

CBCVF_Random10

Elijo esta opción

CBCVF_Random10

Elijo esta opción

Atrás Siguiente

0% 100%

CBCVF_Random11

Imagina que puedes acceder a diferentes opciones de **PrEP**. A continuación te mostramos 12 preguntas donde debes escoger entre **2 opciones potenciales**. En cada pregunta **elige cuál te interesaría más**.

NOTA: El botón para escoger está en la parte inferior. Luego de escoger tu opción preferida, presiona el botón "Siguiente".

(11 of 12)

	Opción A	Opción B
Tipo de PrEP	**Implante** (subcutáneo) 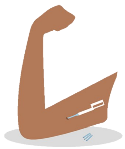	**Inyectable** 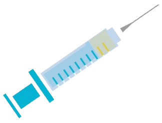
Frecuencia de uso	**1 vez cada 6 meses** 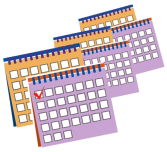	**1 vez al mes** 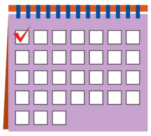
Proveedor de PrEP	**Sector Privado Con costo Menor espera** 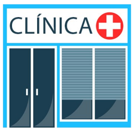	**Sector Privado Con costo Menor espera** 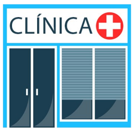
Frecuencia de prueba de VIH	**Prueba de VIH cada 6 meses** 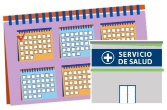	**Prueba de VIH cada 3 meses** 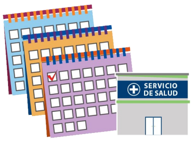
Efectos secundarios	**Sin efectos secundarios** 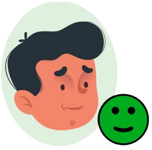	**Leves y permanecen durante semanas** 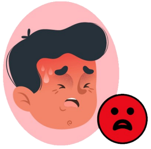
Nivel de eficacia	**El 90% que usa PrEP sigue siendo VIH negativo** 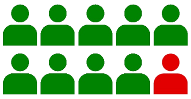	**El 90% que usa PrEP sigue siendo VIH negativo** 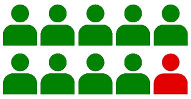

CBCVF_Random11

Elijo esta opción

CBCVF_Random11

Elijo esta opción

Atrás

Siguiente

0% 100%

CBCVF_Random12

Imagina que puedes acceder a diferentes opciones de **PrEP**. A continuación te mostramos 12 preguntas donde debes escoger entre **2 opciones potenciales**. En cada pregunta **elige cuál te interesaría más**.

NOTA: El botón para escoger está en la parte inferior. Luego de escoger tu opción preferida, presiona el botón "Siguiente".

(12 of 12)

	Opción A	Opción B
Tipo de PrEP	**Implante** (subcutáneo) 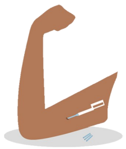	**Oral** 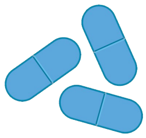
Frecuencia de uso	**1 vez cada 6 meses** 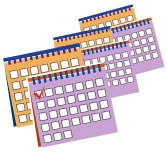	**PrEP por evento (2-1-1)** 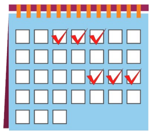
Proveedor de PrEP	**ONG** Costo mínimo Menor espera 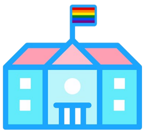	**ONG** Costo mínimo Menor espera 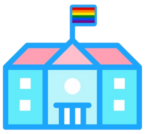
Frecuencia de prueba de VIH	**Prueba de VIH cada 6 meses** 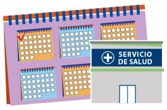	**Prueba de VIH cada 3 meses** 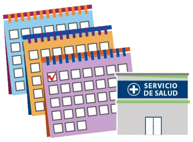
Efectos secundarios	**Leves y permanecen durante semanas** 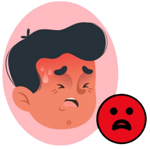	**Sin efectos secundarios** 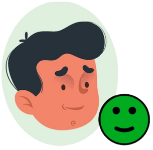
Nivel de eficacia	El **80%** que usa PrEP sigue siendo VIH negativo 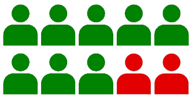	El **70%** que usa PrEP sigue siendo VIH negativo 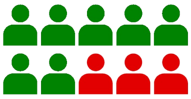

CBCVF_Random12

Elijo esta opción

CBCVF_Random12

Elijo esta opción

Atrás Siguiente

0% 100%

Q27

Considerando tus prácticas sexuales, en tu opinión ¿cuál sería tu riesgo de contraer el VIH **durante los próximos 12 meses**?

Q27=1 Ninguno

Q27=2 Riesgo bajo

Q27=3 Algo de riesgo - 50%

Q27=4 Alto riesgo

Q27=5 Estoy seguro(a) de que lo voy a contraer - 100%

Q27=6 No quiero responder

Atrás Siguiente

0% 100%

Q28

En los últimos 6 meses, ¿tuviste relaciones sexuales con (marca todos los que apliquen):

Q28_1
- [ ] Hombre/Masculino

Q28_2
- [ ] Hombre trans

Q28_3
- [ ] Mujer

Q28_4
- [ ] Mujer trans

Q28_5
- [ ] Travesti

Q28_6
- [ ] No binario

Q28_7
- [ ] No he tenido sexo con nadie en los últimos 6 meses

Q28_8 Q28_8_other
- [ ] Otro (especifique) ____________

Atrás Siguiente

0% 100%

Q29

En los últimos 6 meses, ¿con cuántas personas has tenido sexo?

- Q29=1 Ninguno
- Q29=2 1 persona
- Q29=3 De 2 a 5 personas
- Q29=4 De 6 a 10 personas
- Q29=5 Más de 10 personas
- Q29=6 No sé / No quiero responder

Q31

En los últimos 6 meses, en promedio, ¿cuántos días a la semana has tenido sexo?

- Q31=1 Nunca
- Q31=2 1 día
- Q31=3 2 días
- Q31=4 3-4 días
- Q31=5 5-6 días
- Q31=6 7 (todos los días)
- Q31=7 No quiero responder

Q32

Por favor, contesta las siguientes preguntas sobre tus prácticas sexuales en los últimos 6 meses:

	Sí	No	No quiero responder
¿Has tenido sexo anal pasivo (receptivo) sin condón?	Q32_r1=1	Q32_r1=2	Q32_r1=3
¿Has tenido sexo anal activo (insertivo) sin condón?	Q32_r2=1	Q32_r2=2	Q32_r2=3
¿Has tenido sexo bajo los efectos del alcohol?	Q32_r3=1	Q32_r3=2	Q32_r3=3
¿Has tenido sexo bajo el	Q32_r4=1	Q32_r4=2	Q32_r4=3

efecto de alguna droga?

Q33
En los últimos 6 meses, ¿con cuántas personas tuviste sexo y desconocías su estatus de VIH?

Q33=1 Ninguno

Q33=2 De 1 persona

Q33=3 De 2 o más personas

Q34
En los últimos 6 meses, ¿cuántas de tus parejas sexuales fueron HIV-positivos?

Q34=1 Ninguno

Q34=2 1 persona

Q34=3 2 o más personas

Q34=4 No sé

Q35
En los últimos 6 meses, ¿Cuántas veces tuviste sexo anal insertivo (activo) sin condón con una pareja sexual VIH positivo(a)?

Q35=1 Ninguna

Q35=2 De 1 a 4 veces

Q35=3 De 5 veces a más

Q35=4 No sé

Atrás Siguiente

0% 100%

Q14
Marque abajo todas las drogas que has utilizado en los últimos 6 meses:

Q14_1 Tabaco

Q14_2 Cocaína

Q14_3 Pasta básica

Q14_4 Crack

Q14_5 Éxtasis, MDMA, Molly

Q14_6 GHB/GBL

Q14_7 Special K o ketamina

Q14_8 Metanfetaminas

Q14_9 Poppers

Q14_10 Marihuana

Q14_11 Alucinógenos (LSD, Ayahuasca, hongos, peyote)

Q14_12 Heroína

Q14_13 Inhalantes (ej. Terokal)

Q14_14 Potenciadores de la erección (ej. Viagra. Cialis, Levitra)

Q14_15 No he utilizado estas sustancias en los últimos 6 meses

Q14_16 Q14_16_other
Otro. ¿Cuál?

Q15
En los últimos 6 meses, ¿alguna vez has tomado 5 o más cervezas personales, o 5 o más copas de vino, o 5 o más tragos fuertes (ej. pisco, whisky, ron) en menos de 2 horas?

Q15=1 Sí

Q15=2 No

Q15=3 No quiero responder

Q17

En los últimos 6 meses, ¿con qué frecuencia utiliza las redes sociales (es decir, Facebook, Twitter, etc.), aplicaciones (Grindr, Hornet, Scruff, etc.) o sitios web para buscar parejas sexuales?

Q17=1 Nunca

Q17=2 Una vez al mes

Q17=3 Una vez por semana

Q17=4 En los fines de semana

Q17=5 Diariamente

Q17=6 No quiero responder

Q18

En los últimos 6 meses, ¿has recibido dinero, regalos, vivienda u otro bien a cambio de sexo?

Q18=1 Sí

Q18=2 No

Q18=3 No quiero responder

Atrás Siguiente

0% 100%

Q19

En los últimos 6 meses, ¿has tenido una ITS (infección de transmisión sexual)?

- Q19=1 Sí
- Q19=2 No
- Q19=3 No quiero responder

Atrás Siguiente

0% 100%

Q20

¿Qué ITS te diagnosticaron? (Marque todo lo que corresponda)

Q20_1 ☐ Sífilis

Q20_2 ☐ Clamidia

Q20_3 ☐ Gonorrea

Q20_4 ☐ Herpes genital

Q20_5 ☐ Tricomoniasis

Q20_6 ☐ Chancroide

Q20_7 ☐ Hepatitis B

Q20_8 ☐ Tuve una ITS pero no hice un diagnóstico

Q20_9 ☐ Q20_9_other Otro (especifique) ____________

Q21

¿Tomaste algún medicamento para tratar esta ITS?

Q21=1 ◯ Sí

Q21=2 ◯ No

Q21=3 ◯ No quiero responder

Atrás Siguiente

0% 100%

q58

Durante las últimas 4 semanas: (no hay respuestas correctas o incorrectas)

	Siempre	Casi siempre	Muchas veces	Algunas veces	Solo alguna vez	Nunca
¿Cuánto tiempo estuviste muy nervioso(a)?	q58_r1=1	q58_r1=2	q58_r1=3	q58_r1=4	q58_r1=5	q58_r1=6
¿Cuánto tiempo te sentiste tan bajo(a) de moral que nada podía animarte?	q58_r2=1	q58_r2=2	q58_r2=3	q58_r2=4	q58_r2=5	q58_r2=6
¿Cuánto tiempo te sentiste calmado(a) y tranquilo(a)?	q58_r3=1	q58_r3=2	q58_r3=3	q58_r3=4	q58_r3=5	q58_r3=6
¿Cuánto tiempo te sentiste desanimado(a) y triste?	q58_r4=1	q58_r4=2	q58_r4=3	q58_r4=4	q58_r4=5	q58_r4=6
¿Cuánto tiempo te sentiste feliz?	q58_r5=1	q58_r5=2	q58_r5=3	q58_r5=4	q58_r5=5	q58_r5=6

Atrás Siguiente

0% 100%

q59

Durante las últimas 2 semanas, ¿con qué frecuencia te han molestado los siguientes problemas? (no hay respuestas correctas o incorrectas)

	Ningún día	Varios días	Más de la mitad de los días	Casi todos los días
Poco interés o placer en hacer cosas	q59_r1=1 ◯	q59_r1=2 ◯	q59_r1=3 ◯	q59_r1=4 ◯
Sentirme deprimido(a) o sin esperanza	q59_r2=1 ◯	q59_r2=2 ◯	q59_r2=3 ◯	q59_r2=4 ◯

Atrás Siguiente

0% 100%

q57

Las siguiente oraciones evalúan la perseverancia o persistencia. En una escala del 1 al 5, donde ***1 = Nada parecido a mi*** y donde ***5 = Muy parecido a mi*** , ¿qué tan bien te describen las siguientes oraciones? *No hay respuestas correctas o incorrectas, solo tu opinión.*

	Nada parecido a mi (1)	No muy parecido a mi	Algo parecido a mi	Mayormente parecido a mi	Muy parecido a mi (5)
Nuevas ideas y proyectos a veces me distraen de las ideas y proyectos previos	q57_r1=1 ○	q57_r1=2 ○	q57_r1=3 ○	q57_r1=4 ○	q57_r1=5 ○
Los contratiempos no me desaniman	q57_r2=1 ○	q57_r2=2 ○	q57_r2=3 ○	q57_r2=4 ○	q57_r2=5 ○
He estado obsesionado(a) con alguna idea o proyecto por periodos cortos de tiempo, pero después pierdo el interés	q57_r3=1 ○	q57_r3=2 ○	q57_r3=3 ○	q57_r3=4 ○	q57_r3=5 ○
Soy muy trabajador(a)	q57_r4=1 ○	q57_r4=2 ○	q57_r4=3 ○	q57_r4=4 ○	q57_r4=5 ○
Con frecuencia me fijo una meta, pero después escojo otra diferente	q57_r5=1 ○	q57_r5=2 ○	q57_r5=3 ○	q57_r5=4 ○	q57_r5=5 ○
Tengo dificultad para enfocarme en proyectos que toman más de unos meses para terminarlos	q57_r6=1 ○	q57_r6=2 ○	q57_r6=3 ○	q57_r6=4 ○	q57_r6=5 ○
Termino todo lo que empiezo	q57_r7=1 ○	q57_r7=2 ○	q57_r7=3 ○	q57_r7=4 ○	q57_r7=5 ○
Soy diligente, es decir, hago las cosas de manera exacta y cuidadosa	q57_r8=1 ○	q57_r8=2 ○	q57_r8=3 ○	q57_r8=4 ○	q57_r8=5 ○

Atrás   Siguiente

0% 100%

Q60

¡Muchas gracias por completar la encuesta!

Te invitamos a revisar mayor información sobre PrEP haciendo clic en los siguientes botones de OMS, OPS e ImPrEP:

0% 100%
